# The Neurexin1β Histidine-Rich Domain Is Involved in Excitatory Presynaptic Organization and Short-Term Plasticity

**DOI:** 10.1523/ENEURO.0399-25.2026

**Published:** 2026-01-30

**Authors:** Benjamin Feller, Mai Inagaki, Manni Wang, Annika Sivak, Nicolas Chofflet, Hideto Takahashi

**Affiliations:** ^1^Synapse Development and Plasticity Research Unit, Institut de Recherches Cliniques de Montréal, Montreal, Quebec H2W 1R7, Canada; ^2^Department of Neuroscience, Faculty of medicine, Université de Montréal, Montreal, Quebec H3T 1J4, Canada; ^3^Graduate School of Biomedical Sciences, Tokushima University, Tokushima, 770-8505, Japan; ^4^Integrated Program in Neuroscience, McGill University, Montreal, Quebec H3A 2B2, Canada; ^5^Department of Molecular Biology, Faculty of Medicine, Université de Montréal, Montreal, Quebec H3T 1J4, Canada; ^6^Division of Experimental Medicine, McGill University, Montreal, Quebec H3A 0G4, Canada

**Keywords:** histidine-rich domain, neurexin1β, neuroligin 1, short-term presynaptic plasticity, synapse development, synaptic transmission

## Abstract

Neurexins (Nrxns) are presynaptic cell adhesion molecules essential for synapse development and function. Of the many neurexin isoforms, only β-Nrxns contain the histidine-rich domain (HRD). While the HRD has been implicated in several pathological contexts, its normal physiological role remains unclear. To address this, we used a CRISPR-Cas9 method to generate a new mouse line expressing in-frame truncated Nrxn1β lacking the HRD. We found that HRD deletion did not affect mouse viability, gross brain development, or general behavior of either sex. However, loss of the HRD significantly altered neuroligin-1-dependent excitatory, but not inhibitory, presynaptic differentiation in primary cultured neurons. Moreover, this deletion affected presynaptic short-term plasticity, but not basal synaptic transmission, at hippocampal Schaffer collateral→CA1 synapses. These findings identify the Nrxn1β HRD as a potential contributor to excitatory presynaptic organization and function, providing new insight into the molecular diversity and specialization of Nrxns.

## Significance Statement

The histidine-rich domain (HRD) is a unique domain of β-Nrxns, absent from α-Nrxns, that mediates the binding of pathological proteins. This study investigates the physiological functions of the Nrxn1β HRD by generating and characterizing a new mouse line expressing a truncated *Nrxn1β* mutant lacking its HRD. We found that Nrxn1β HRD deletion affects paired-pulse facilitation, a form of short-term presynaptic plasticity, and neuroligin-1-mediated excitatory presynaptic organization without affecting overall brain development, synapse formation, basal synaptic transmission, or mouse behavior. These findings highlight the critical role of Nrxn1β HRD in excitatory presynaptic organization and function. This work will open new avenues for studying isoform-specific mechanisms of Nrxns for synaptic regulation and their potential implications in both physiological and pathological contexts.

## Introduction

The neurexins (Nrxns) form a family of presynaptic cell adhesion molecules that play an essential role in synapse assembly, maturation, and function in the central nervous system (Sudhof, 2017; Gomez et al., 2021). The functional heterogeneity of Nrxns in mammals comes from their large number of isoforms, which arise from three distinct genes (*Nrxn1–3*), each containing two separate promoters (α and β) that independently regulate the production of α- and β-Nrxns alongside several canonical alternative splicing sites (SS1 to SS6; Lukacsovich et al., 2019). Distinctive characteristics of the various isoforms are believed to serve as a molecular code, tightly regulating the Nrxn interactome and thereby shaping local synaptic properties (Sudhof, 2017). For instance, the predominant isoform of neuroligin 1 (Nlgn1), a well-characterized postsynaptic ligand of Nrxns, interacts with β-Nrxns, but not α-Nrxns (Boucard et al., 2005), to promote excitatory, but not inhibitory, presynaptic differentiation (Chih et al., 2006). In contrast, neurexophilin1 (Nxph1), an α-Nrxn-specific ligand, is involved in inhibitory short-term plasticity (Born et al., 2014), thus emphasizing the layered complexity of Nrxn functions. Emerging evidence has started to delineate the distinct physiological roles of each Nrxn (Boxer et al., 2021; Lin et al., 2023), but, in contrast to their α-Nrxn counterparts, the specific contributions of β-Nrxns remain poorly understood.

**Figure 1.**
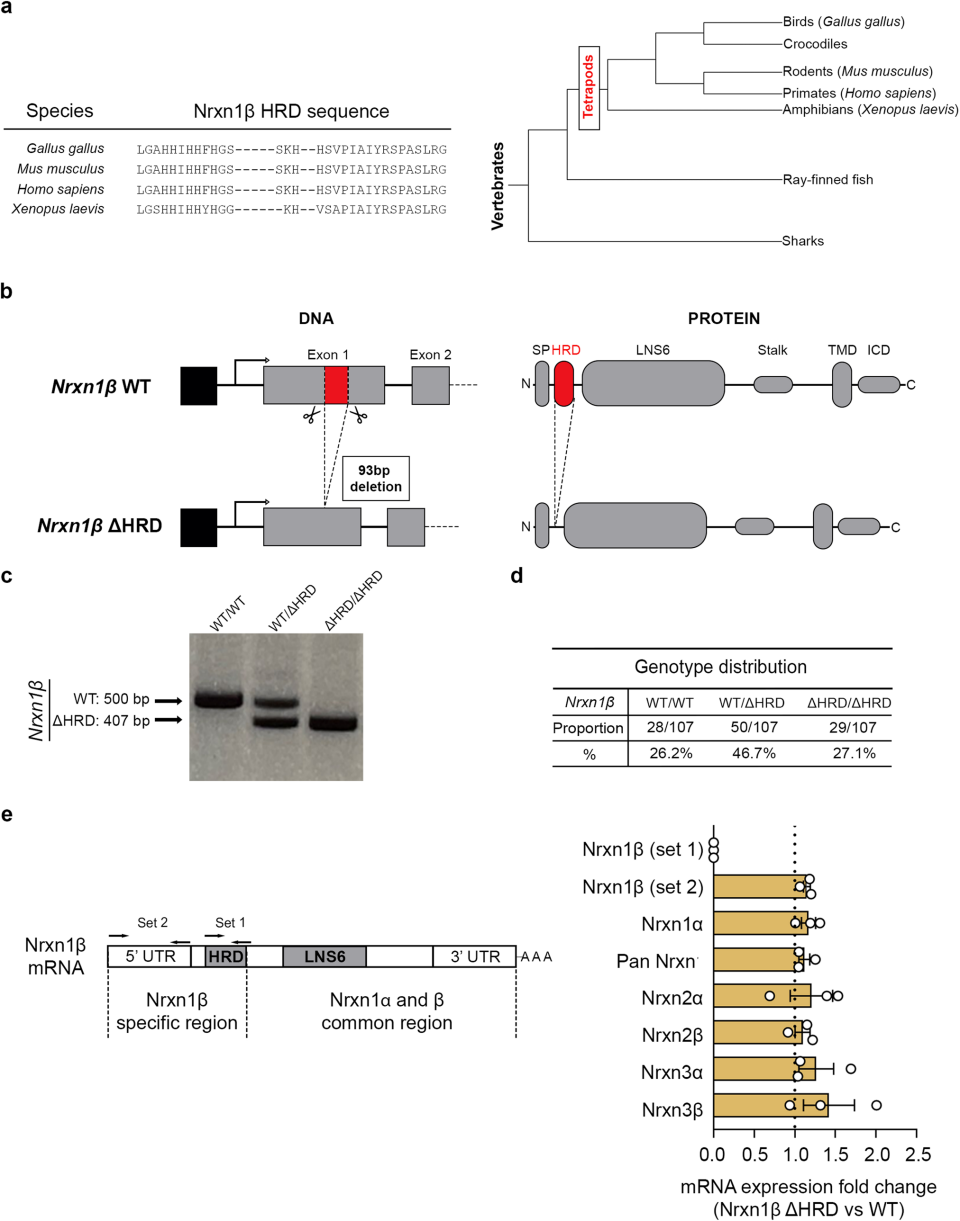
*Nrxn1β* gene modification strategy and expression verification. ***a***, Comparison of the HRD sequence within the Nrxn1β protein across different vertebrate species. ***b***, Illustration representing the modification of the *Nrxn1β* gene. Using CRISPR-CAS9 genome editing, the 93 bp coding sequence for the HRD was removed from exon 1 of *Nrxn1β*. ***c***, Verification of HRD deletion at the DNA level by genotyping. The non-modified *Nrxn1β* amplified sequence is 500 bp long while the sequence amplified in *Nrxn1β* ΔHRD mice is 407 bp. The exact deleted sequence is presented in Extended Data Fig. 1-1. ***d***, Evaluation of genotype distribution throughout generations. Data comes from the evaluation of progeny from 10 heterozygous *Nrxn1β* ΔHRD breeding pairs (*n* = 107 mice). ***e***, Left, Representation of the *Nrxn1β* mRNA regions. “Set 1” indicates the primers targeting specifically the HRD of Nrxn1β to confirm its deletion at the mRNA level. “Set 2” indicates the primers targeting both *Nrxn1β* WT and *Nrxn1β* ΔHRD (but not *Nrxn1α*) to evaluate the effect of HRD deletion on *Nrxn1β* mRNA expression. Right, Quantification of the fold change in hippocampal mRNA expression of several genes of interest from *Nrxn1β* ΔHRD mice compared with *Nrxn1β* WT using RT-qPCR. Expression fold changes were obtained using a 2^−ΔΔC_T_^ transformation. Data are presented as mean ± SEM. *n* = 4/3 mice (WT/ΔHRD).

Structurally, α-Nrxns are type I transmembrane proteins characterized by an extracellular domain composed of six laminin-neurexin-sex hormone-binding globulin (LNS) domains (LNS1–6) interspersed with three epidermal growth factor (EGF)-like domains (EGF1–3), followed by a carbohydrate attachment site (CHO), a transmembrane domain, and an intracellular cytoplasmic tail containing a PDZ domain-binding motif (Sudhof, 2017). In contrast, β-Nrxns represent a shorter version of α-Nrxns, sharing only the terminal part ranging from LNS6 to the C-terminal cytoplasmic tail. However, β-Nrxns also possess a distinguishing N-terminal region comprising an unusually long signal peptide sequence and a histidine-rich domain (HRD; Reissner et al., 2013). Evolutionarily, β-Nrxns and their HRD seem to originate with vertebrates; invertebrates, such as *Drosophila melanogaster* and *Caenorhabditis elegans*, do not express any β-Nrxn isoform (Tabuchi and Sudhof, 2002). This suggests that the β-Nrxn HRD may have evolved to fulfill specialized functions in species with more complex central nervous systems, such as tetrapods, which display a highly conserved HRD (Fig. 1*a*).

10.1523/ENEURO.0399-25.2026.f1-1 Figure 1-1 **Confirmation of the mouse line in-frame Nrxn1β HRD deletion through sequencing** Following genotyping, the WT and ΔHRD bands from a heterozygous *Nrxn1β* ΔHRD mouse (see Fig. 1c) were excised from the agarose gel and cloned into a plasmid for sequencing. Sequencing confirmed the deletion of the 93 bp region (highlighted bases) encoding the HRD in the mature protein. Download Figure 1-1, TIF file.

The functionality of the HRD may be related to the distinctive properties of histidine imidazole side chains which can reversibly switch between protonated and neutral states near physiological pH. This dual property is essential in transient interactions, including protein structure stabilization and protein–protein or protein–metal binding (Calinsky and Levy, 2024). Such features may be particularly relevant to β-Nrxn functions: they may allow for dynamic regulation of ligand interactions within the fast-changing environment of the synaptic cleft. Our group has previously demonstrated important roles for the β-Nrxn HRD in pathological contexts. This domain mediates interactions between β-Nrxns and pathogenic ligands such as amyloid-β oligomers implicated in Alzheimer's disease as well as α-synuclein preformed fibrils associated with Parkinson's disease (Naito et al., 2017; Feller et al., 2023). Additionally, another study has discovered that all β-Nrxns bind the bacterial adhesin matrix molecule SrdC through their HRD (Barbu et al., 2010). Despite the documented pathological relevance of the HRD, its normal physiological role remains largely unexplored. To address this, we used a CRISPR-Cas9 genome editing approach to generate a novel mouse model expressing an in-frame truncated Nrxn1β mutant specifically lacking the HRD.

Here, we provide a comprehensive characterization of this mouse model, examining its molecular expression profile, synaptic effects, and potential behavioral repercussions. We found that deletion of the Nrxn1β HRD has no significant effects on overall mouse health, gross brain development, or mouse behavior. However, we discovered that HRD deletion has significant impacts on Nlgn1-induced excitatory, but not inhibitory, presynaptic differentiation as well as presynaptic short-term plasticity of Schaffer collateral excitatory synapses. Together, these data highlight the specific contribution of the Nrxn1β HRD to excitatory presynaptic organization and function.

## Material and Methods

### Mouse generation

Deletion of the *Nrxn1β* HRD coding sequence (ΔHRD) was performed using a CRISPR/Cas9 method. Specific gRNA targeting *Nrxn1β* (gRNA1: GATGTGGTGCGCTCCCAAACTGG/gRNA2: TCCTTGCGAGGCGGACACGGTGG) and Cas9 mRNA were coinjected into fertilized C57BL/6J mouse eggs to generate targeted knock-out offspring. F0 founder animals were identified by PCR followed by sequence analysis and bred to wild-type mice to test germline transmission and F1 animal generation. The positive F1 animals were then used for subsequent backcrossing with C57BL/6J, and all experiments were performed after at least 10 backcrossings.

### Genotyping

Genotyping of Nrxn1β ΔHRD mice was performed by extracting DNA from mouse tail. Tails were digested by adding 120 µl of 50 mM NaOH and heating the mixture at 98°C for 1 h. A total of 30 µl of 1 M HCl, pH 7.4, were then added to neutralize the reaction. PCR amplification was carried out using the following reaction mix: 2 µl of 10× Qiagen buffer (catalog #201223, Qiagen), 0.4 µl of dNTP mix (catalog #201900, Qiagen), 1 µl of forward primer at 10 µM (5′-TCTTCTCTTCTTGTGCCCCAAAC-3′), 1 µl of reverse primer at 10 µM (5′-GCCTTCACCCATCCCTTTCTATTG-3′), 0.05 µl of Taq DNA polymerase at 5 U/µl (catalog #201223, Qiagen), and 0.5 µl of the previously extracted DNA. PCR was performed using a Mastercycler Nexus Gradient GSX1 Thermal Cycler (catalog #8125-30-1027, Eppendorf) under the following conditions: 94°C for 2 min, (94°C for 30 s, 61°C for 30 s, 72°C for 90 s) ×35cycles and 72°C for 7 min. After the PCR, 4 µl of 6× gel loading dye (catalog #B7024S, New England Biolabs) was added to the PCR product, and the samples were loaded onto a 2% agarose gel containing 0.004% ethidium bromide and run at 100 V for 40 min. DNA bands were visualized using a UVP BioDoc-It imaging system (catalog #17-1718, Taylor Scientific). Expected PCR product sizes are 500 bp for the wild-type (WT) allele and 407 bp for the ΔHRD mutant allele.

### Mouse brain dissection

**Figure 2.**
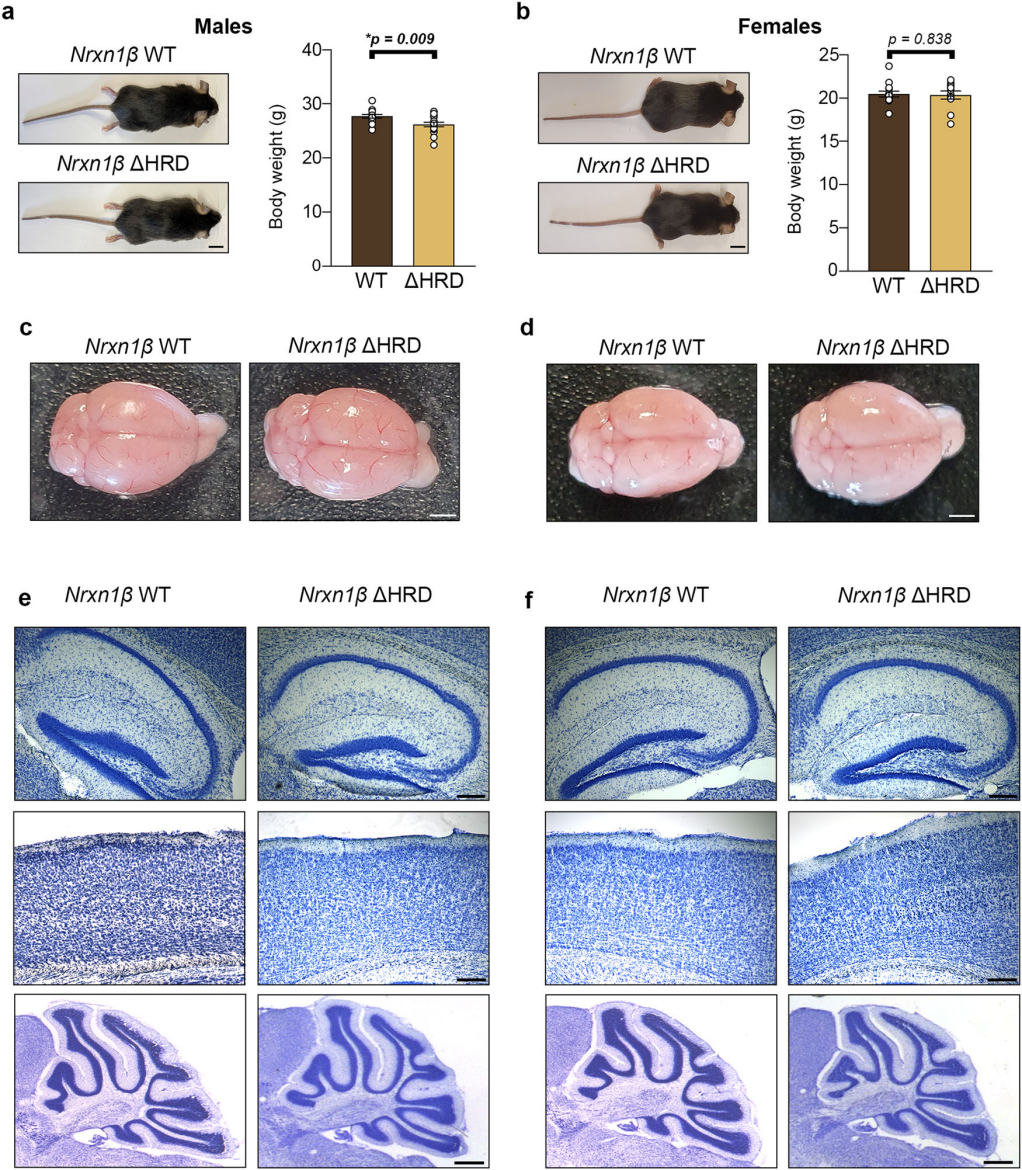
Assessment of body weight and brain morphology in adult *Nrxn1β* ΔHRD mice. ***a***, Left, Photography of *Nrxn1β* WT and *Nrxn1β* ΔHRD adult males. Right, Body weight measurement of adult *Nrxn1β* WT and *Nrxn1β* ΔHRD males. Average age = 73.27 ± 7.91/73.06 ± 7.93 (WT/ΔHRD) days. Statistical significance was examined by unpaired *t* tests. Results are presented as mean ± SEM and *n* = 15/17 mice (WT/ΔHRD). Scale bar, 1 cm. ***b***, Left, Photography of *Nrxn1β* WT and *Nrxn1β ΔHRD* adult females. Right, Body weight measurement of adult *Nrxn1β* WT and *Nrxn1β* ΔHRD females. Average age = 77.71 ± 14.18/76.45 ± 15.69 (WT/ΔHRD) days. Statistical significance was examined by unpaired *t* tests. Results are presented as mean ± SEM and *n* = 14/11 mice (WT/ΔHRD). Scale bar, 1 cm. ***c***, ***d***, Photography of *Nrxn1β* WT and *Nrxn1β* ΔHRD male and female brains, respectively. Scale bar, 20 mm. ***e***, ***f***, Light microscopy imaging of Nissl-stained sagittal brain slices from male (***e***) and female (***f***) *Nrxn1β* WT and *Nrxn1β* ΔHRD mice showing brain regions in which Nrxn1β is known to be strongly expressed (hippocampus, cortex, and cerebellum; Uchigashima et al., 2019). Scale bar, 200 µm for hippocampus/cortex and 1 mm for cerebellum.

All animal procedures were conducted in accordance with the Canadian Council on Animal Care guidelines and approved by the IRCM Animal Care Committee (project numbers: 2021-1116 and 2025-1289). Mice were deeply anesthetized using a mix of intraperitoneally injected ketamine/xylazine (10 mg/ml ketamine, 1 mg/ml xylazine, in 0.9% saline; dose: 0.1 ml per 10 g of body weight) and killed using a CO_2_ chamber. The absence of pedal reflexes was confirmed prior to decapitation and brain extraction, which was performed as previously described (Aboghazleh et al., 2024). Brains were either immediately imaged for morphological characterization (Fig. 2) or further dissected to isolate regions of interest such as the hippocampus or cortex for reverse transcription-quantitative PCR (RT-qPCR) or proteomic analysis. Dissected tissues were transferred into 1.5 ml Eppendorf tubes, snap-frozen in liquid nitrogen, and stored at −80°C until use.

### Nissl staining

WT and homozygous *Nrxn1β* ΔHRD mice (*Nrxn1β* ΔHRD) at 3 months old were deeply anesthetized using a mix of intraperitoneally injected ketamine/xylazine (10 mg/ml ketamine, 1 mg/ml xylazine, in 0.9% saline; dose: 0.1 ml per 10 g of body weight) and after confirmation of pedal reflex absence, mice were transcardially perfused with PBS followed by 4% paraformaldehyde (PFA) as described previously (Khaled et al., 2024). Brains were dissected, postfixed overnight in 4% PFA, then washed in PBS, and transferred to a 30% sucrose solution for 48 h for cryoprotection. Subsequently, brains were embedded in OCT compound (catalog #23-730-571, Fisher Scientific) and stored at −80°C for at least 24 h before cryosectioning. Sagittal sections (50 µm thick) were obtained using a cryostat and collected in 12-well plates containing PBS. Brain slices were then placed onto Premium Superfrost Plus Microscope slides (catalog #CA48311-703, VWR) and air-dried at room temperature for an hour. Next, slides were incubated overnight in 1:1 ethanol/chloroform solution. The slides were rehydrated in 100, 95, and 70% ethanol baths the following day. Nissl staining was performed by immersing slides in 0.1% cresyl violet solution for 10 min at room temperature, rinsing three times in distilled water, and differentiating in 95% ethanol. Slides were then dehydrated in 100% ethanol, cleared in xylene, and mounted with mounting medium. Images of the cerebellum were taken using a Leica Stereo Microscope MZ12 while hippocampal and cortical regions were imaged using a Leica DM6 fluorescent microscope at 5× magnification.

### RT-qPCR

RNA was extracted from seven previously collected 4-month-old female mouse hippocampi (see above, Mouse brain dissection) using an RNeasy mini kit (catalog #74104, Qiagen) following the manufacturer's instructions. Total RNA concentration was determined by measuring the absorbance at 260 nm (A260) with a NanoDrop spectrophotometer (catalog #8353-30-0010, Thermo Scientific). Total RNA purity was verified by determining the A260/A280 ratio. For each sample, a 20 μl mix containing 2 μg total RNA, 150 ng of random hexamers, and 1 μl of a 10 mM dNTP was prepared. Then reverse transcription was performed in an Eppendorf Mastercycler with an initial priming step at 25°C for 10 min, followed by cDNA synthesis at 37°C for 120 min. A final inactivation step at 85°C for 5 min completed the reaction. Wells of a 384-well plate were loaded with 3 µl of cDNA (2 ng/µl) and 7 µl of a mix containing 1× PowerUp SYBR Green Master Mix for qPCR (catalog #A25741, Invitrogen) and primer sets (Table 1) diluted to 1.5 µM in DNAse-free water. Each sample was loaded in triplicate, and RT-qPCR was performed using a QuantStudio 5 Real-Time PCR system. Data were analyzed with Design and Analysis qPCR 2.8.0 software, and fold changes were calculated as previously described (Livak and Schmittgen, 2001).

**Table 1.** List of primers used for RT-qPCR

Primer name	5′ → 3′ sequence
Pan-Nrxn1 (Fwd)	GGATAGTCCCGCTCACCC
Pan-Nrxn1 (Rev)	TCTGTCCACCACCTTTGC
Nrxn1α (Fwd)	GCAAAACCGTGCCTTAGCAA
Nrxn1α(Rev)	TCACAGGACCTGCCGAGATA
Nrxn1β (Set 1) (Fwd)	AAGCATCATTCAGTGCCTATTG
Nrxn1β (Set 1) (Rev)	GGCCACTTATATGTAATCTGTC
Nrxn1β (Set 2) (Fwd)	ACTCTCGCCCGAAACTCTTG
Nrxn1β (Set 2) (Rev)	GTGCCCTACAACAGTACCCC
Nrxn2α (Fwd)	CCTGCGCGCAATCCCT
Nrxn2α (Rev)	GTGAGCCGGACCTTCCAT
Nrxn2β (Fwd)	CACTTCCACAGCAAGCAC
Nrxn2β (Rev)	CTTCCCGAAGATGTATGTG
Nrxn3α (Fwd)	GCCACACTGGATCCCATCAA
Nrxn3α (Rev)	AAGAGAATCAGGCCGTTGGG
Nrxn3β (Fwd)	CGTCAGCTCTGTGTGCAGTT
Nrxn3β (Rev)	TAGGCACAGAGTGGTGCTTG
ACTB (Fwd)	CCTGAACCCTAAGGCCAACC
ACTB (Rev)	ATGGCGTGAGGGAGAGCATA

This table gives sequence details about the primers used for RT-qPCR experiments. Pairs of forward (Fwd) and reverse (Rev) primers are both given in their 5′–3′ orientation.

### Primary neuron culture

Primary cortical neuron cultures were prepared from embryonic day 18 (E18) mouse embryos obtained from heterozygous *Nrxn1β* ΔHRD matings. Briefly, after killing the dam using CO_2_, embryos were extracted and placed in a 10 cm Petri dish. After decapitation of the embryos, embryonic brains were extracted and dissected, and their cortices were placed into a numbered 12-well plate containing Hibernate E (catalog #NC0285514, Fisher Scientific) and stored at 4°C. Meanwhile, genotyping was performed on the tails from the same embryos (see above, Genotyping). One WT and one mutant cortex were then selected for culture and transferred to separate 15 ml tubes each containing 2 ml of papain solution [100 mM CaCl_2_, 50 mM EDTA, pH 8.0, cysteine crystals, and 20 U/ml of papain (catalog #LS003119, Worthington) dissolved in sterile calcium- and magnesium-free Hanks balanced salt solution (CMF-HBSS)] and incubated at 37°C in 5% CO_2_ atmosphere for 20 min. The cells were pelleted by centrifugation at 100*×g* for 4 min, and the papain solution supernatant was removed and replaced with 2 ml of plating medium [0.6% glucose, 10% horse serum, 1× HyClone Penicillin/streptomycin solution (catalog #SV30010, Cytiva) in MEM (catalog #11095098, Thermo Fisher Scientific)] containing 2.5 mg/ml of bovine serum albumin and 2.5 mg/ml of trypsin inhibitor to inactivate the digestion. The tissues were gently triturated using a P1000 pipette until a homogenous solution was obtained. Dissociated cortical neurons were plated onto five coverslips placed in 6 cm culture dishes, at a density of 2 million neurons per dish. Twenty-four hours after plating, coverslips were transferred to another 6 cm dish containing a glial feeder layer. Neurons were maintained until 21 d in vitro (DIV) by replacing 50% of the medium once or twice a week.

### Plasmids

Plasmids encoding extracellular HA-tagged mouse neuroligin 1 lacking splice site A and containing splice site B (HA-Nlgn1(B+) or mouse neuroligin 2 lacking splice site A (HA-Nlgn2) were kindly provided by Dr. Peter Scheiffele through Addgene (Plasmid #15261 and #15246). Plasmids expressing extracellular HA-tagged CD4 (HA-CD4), Nrxn1β (HA-Nrxn1β), or Nrxn1β lacking HRD (HA-Nrxn1βΔHRD) were described previously (Lee et al., 2023a). To generate the construct expressing the Nlgn1(B+) ectodomain fused to the human immunoglobulin Fc region [pc4-Nlgn1 (B+)-Fc], the coding sequence of the mature form of the Nlgn1(B+) ectodomain was amplified by PCR using HA-Nlgn1(B+) as a template and subsequently subcloned into pc4-spNRX1β-Fc cloning vector downstream of the NRX1β signal sequence. All constructs were verified by DNA sequencing.

### Artificial synapse formation assay

Artificial synapse formation assays are used to evaluate the synaptogenic activity of surface proteins by coculturing neuronal cells with fibroblasts transfected with synapse-inducing proteins. These experiments were performed as we have done previously (Tanabe et al., 2017; Feller et al., 2023; Chofflet et al., 2024). Briefly, HEK293T (ATCC) cells were first transfected with the indicated extracellular HA-tagged expression vectors that we previously described (Feller et al., 2023) using TransIT-LT1 Transfection Reagent (Mirus Bio). HA-Nlgns were used as a Nrxn-mediated presynapse-inducing proteins while HA-CD4 was used as a negative control, as it does not interact with Nrxns and does not induce presynaptic differentiation. After 24 h, the HEK293T cells were harvested through trypsinization, and 10,000 cells were cocultured with 15 DIV cortical neurons from *Nrxn1β* ΔHRD or WT mice. After a 24 h incubation period, neurons, alongside HEK293T cells, were fixed using parafix solution (4% paraformaldehyde and 4% sucrose in PBS, pH 7.4) for 12 min at room temperature (RT) and blocked using a blocking solution (PBS + 3% BSA and 5% normal donkey serum). To identify surface expression of HA-tagged proteins, cells were treated with a primary antibody directed against HA (1:2,000; mouse IgG2bκ, catalog #11583816001, RRID: AB_514505, MilliporeSigma) overnight at 4°C. Next, cells were permeabilized with PBST (PBS + 0.2% Triton X-100), blocked with the same solution as for the HA immunostaining, and then stained for MAP2, VGLUT1, and VGAT overnight at 4°C using the corresponding primary antibodies: anti-MAP2 (1:2,000; chicken polyclonal IgY; catalog #ab5392, RRID: AB_2138153, Abcam), anti-VGLUT1 (1:250; guinea pig; catalog #135304, RRID: AB_887878, Synaptic Systems), anti-VGAT (1:1,000; rabbit IgG, catalog #131 003, RRID: AB_887869, Synaptic Systems). VGLUT1 and VGAT antibodies are used to label excitatory and inhibitory presynaptic compartments, respectively. MAP2 antibody was used to label neuronal soma and dendrites. Highly cross-adsorbed Alexa dye-conjugated secondary antibodies generated in donkey toward the appropriate species (1:500; AMCA, Alexa488, Alexa594, and Alexa647; Jackson ImmunoResearch) were used as detection antibodies. Images were acquired using a Leica DM6 fluorescent microscope with a 40× magnification objective focused on areas where HA-expressing HEK293T cells were present. The VGLUT1 and VGAT total intensity around the HEK293T cells was calculated after thresholding the background. All analyses were performed under blind conditions. Statistical analysis was performed using a linear mixed-effects model in R, to account for variation between experiments and effectively isolate the effect of genotype. The results include the estimated difference in intensity between Nrxn1β ΔHRD and WT cortical neurons, the calculated *p* value, 95% confidence interval and marginal *R*^2^, which represents the effect size of the fixed effect (genotype).

### Cell surface Nlgn1-Fc protein binding assay

Recombinant Nlgn1-Fc proteins were generated by transfecting HEK293T cells with pc4-Nlgn1 (B+)-Fc using TransIT-PRO Transfection Reagent (catalog #MIR5740; Mirus Bio). Transfected cells were maintained for 3 d in a serum-free AIM V synthetic medium (catalog #12055083; Thermo Fisher Scientific). Afterward, supernatant containing Nlgn1-Fc proteins was harvested and kept at −80°C until use. To assess Nlgn1-Fc binding, COS-7 cells were transfected with HA-Nrxn1β, HA-Nrxn1βΔHRD, or HA-CD4 using TransIT-LT1 Transfection Reagent (catalog #MIR2304; Mirus Bio). After 24 h, transfected cells were washed with an extracellular solution (ECS; 2.4 mM KCl, 2 mM CaCl2, 1.3 mM MgCl2, 168 mM NaCl, 20 mM HEPES, pH 7.4, and 10 mM D-glucose) supplemented with 100 μg/ml of BSA (ECS/BSA), then incubated with Nlgn1-Fc proteins in ECS/BSA for 1 h at 4°C to prevent endocytosis. Cells were washed three times using ECS and then fixed in parafix solution (4% paraformaldehyde and 4% sucrose in PBS, pH 7.4) for 12 min at RT. To immunolabel bound Fc proteins and surface HA, fixed cells were incubated with a blocking solution (PBS containing 3% BSA and 5% normal donkey serum) for 1 h at RT. Without cell permeabilization, cells were incubated with anti-HA (1:2,000; rabbit IgG, catalog #ab9110; Abcam) in the blocking solution overnight at 4°C, followed by secondary antibodies of donkey Alexa488-conjugated anti-rabbit IgG (H + L) and donkey Alexa594-conjugated anti-human IgG (H + L) for 1 h at RT. Images were acquired using a Leica DM6 fluorescent microscope with a 40× objective. Statistical analysis was performed using a linear mixed-effects model in R, to account for interexperiment variability and isolate construct effects.

### Synaptic density assessment

Synapse density was assessed using 15 DIV cortical neurons from *Nrxn1β* ΔHRD and WT mice. After fixation with parafix solution, neurons were permeabilized by treatment with PBST for 5 min and blocked using the same blocking solution as above. To specifically identify excitatory synapses, antibodies against VGLUT1 (1:250; guinea pig; catalog #135304, RRID: AB_887878, Synaptic Systems) and PSD95 (1:1,000; mouse IgG2a; catalog #MA1-045, RRID: AB_325399, Thermo Fisher Scientific) were used. To specifically identify inhibitory synapses, antibodies against VGAT (1:1,000; rabbit IgG; catalog #131 003, RRID:AB_887869, Synaptic Systems) and Gephyrin (1:500; mouse IgG1; catalog #147011, RRID: AB_2810215, Synaptic Systems). An antibody against MAP2 was also applied to stain the dendrites. Highly cross-adsorbed Alexa dye-conjugated secondary antibodies generated in donkey toward the appropriate species (1:500; AMCA, Alexa488, Alexa594; Jackson ImmunoResearch) were used as detection antibodies. Images were acquired using a Leica DMi8 confocal microscope with 63× objective and secondary dendrites were targeted for analysis of synapse density. After defining a region of interest and thresholding the background, colocalized VGLUT1 and PSD95 puncta were manually counted. The density was obtained by dividing this number by the dendritic length present in the region of interest. All analyses were performed with the experimenter blind to genotype. Statistical analysis was performed using a linear mixed-effects model in R, to account for variation between experiments and effectively isolate the effect of genotype. The results include the estimated difference in synapse density between Nrxn1β ΔHRD and WT cortical neurons, the calculated *p* value, 95% confidence interval and marginal *R*^2^, which represents the effect size of the fixed effect (genotype).

### Proteomic analysis

Synaptosome fractions were prepared from previously frozen hippocampus and cortex (see above, Mouse brain dissection) from eight (4 males and 4 females) 2-month-old *Nrxn1β* ΔHRD and eight (4 males and 4 females) age-matched WT mice. Briefly, tissues were homogenized in homogenization buffer containing 0.32 M sucrose, 4 mM HEPES, pH 7.4, HPLC-grade H_2_O, and protease inhibitor cocktail (catalog #5892791001, Roche) and centrifuged at 1,000*×g* for 15 min to pellet large debris and nuclei. The supernatant was further centrifuged for 25 min at 14,000*×g* to pellet crude synaptosomes. The pellet was resuspended in urea buffer containing 7 M urea (catalog #97063-802, VWR), 2 M thiourea (catalog #T865 6-50G, Sigma Aldrich), 4% CHAPS (catalog #97063-816, VWR), 25 mM DTT (catalog #M109-5G, VWR), 100 mM EPPS (catalog #J61476.AK, Thermo Fisher Scientific), HPLC-grade H_2_O, and protease inhibitor cocktail (catalog #5892791001, Roche). A quick start Bradford protein assay (catalog #5000201, Bio-Rad) was performed to evaluate the protein concentration, samples were diluted to 1 mg/ml, and 50 µl of each was used for the following steps.

Samples were initially reduced with 20 mM dithiothreitol (DTT) in 100 mM EPPS and incubated on an Eppendorf ThermoMixer at 37°C for 30 min at 700 rpm. After cooling for 5 min, samples were alkylated with 37 mM iodoacetamide (IAA) in 100 mM EPPS at room temperature for 30 min at 700 rpm, protected from light. To remove residual nucleic acids, samples were treated with 50 units of benzonase per sample in the presence of 2 mM MgCl_2_ and incubated at 37°C for 60 min at 700 rpm. Protein Aggregation Capture (PAC) was performed using 25 µl of washed hydroxyl beads (Resyn Biosciences) per sample. Then, 100% acetonitrile was added to achieve a final concentration of 50%, initiating protein aggregation. Samples were incubated at room temperature for 20 min at 1,000 rpm and then placed on a magnetic rack for 2 min. Supernatants were removed and retained for further analysis. Beads were washed three times with 70% ethanol. Following the final wash, beads were resuspended in 100 µl of Lys-C/Trypsin mix (Promega; final concentration 0.025 µg/µl) in 100 mM EPPS. Samples were briefly sonicated in a water bath for 2 min and digested overnight at 37°C with shaking at 700 rpm. Upon completion of digestion, samples were placed on a magnetic rack, and the supernatants were transferred to Eppendorf LoBind tubes. Beads were rinsed with 50 µl of 100 mM EPPS, and the rinse supernatants were pooled with the initial digests.

Protein digests were acidified with trifluoroacetic acid (TFA) and cleaned of residual reagents using an Oasis HLB extraction plate (Waters), according to the manufacturer's instructions. The purified peptides were lyophilized and reconstituted in 25 µl of 100 mM EPPS buffer with agitation for 15 min. Isobaric labeling was performed using a 16-plex Tandem Mass Tag (TMT) kit (Thermo Fisher Scientific) following the manufacturer's protocol. After labeling, samples were pooled, acidified with TFA, and desalted using a Waters Oasis HLB 96-well elution plate. The pooled and cleaned peptides were then dried by vacuum centrifugation and reconstituted in 20 µl of 2% acetonitrile (ACN)/1% formic acid (FA) with agitation for 15 min. High-pH reversed-phase peptide fractionation was performed using a Pierce High pH Reversed-Phase Peptide Fractionation kit (Thermo Fisher Scientific) according to the manufacturer's instructions. Each TMT-labeled sample was fractionated into 10 fractions. Fractions were dried by vacuum centrifugation and reconstituted in 12 µl 2% ACN/1% FA with agitation for 15 min prior to LC-MS/MS analysis.

Samples were loaded onto a 75 μm i.d. × 250 mm PicoChip C18 column (New Objective) installed in a Vanquish Neo LC system (Thermo Scientific). The buffers used for chromatography were 0.2% formic acid (buffer A) and 85% acetonitrile/0.2% formic acid (buffer B). Peptides were eluted at a 280 nl/min flow rate using a three-step linear gradient: solvent B first increased from 3 to 29% in 108 min, then from 29 to 40% B in 27 min, and finally from 40 to 90% B in 3 min. The LC system was interfaced to an Orbitrap Fusion mass spectrometer (Thermo Scientific) through a PicoChip source (New Objective). Nanospray and S-lens voltages were set to 1.8–2.5 kV and 50 V, respectively. Capillary temperature was set to 250°C. Full scan MS survey spectra (m/z 360-1400) were acquired in the Orbitrap with a resolution of 60,000 and a target value set at 8 × 10^5^. The most intense precursor ions were fragmented via HCD with a normalized collision energy of 38%, and fragment ions were analyzed in the Orbitrap at 50,000 resolution, with an AGC target of 1 × 10^5^. The duty cycle was set to 3 s, and dynamic exclusion was applied for 30 s after two MS2 spectra per precursor.

Raw MS data were searched against the SwissProt database restricted to *Mus musculus* using Proteome Discoverer version 2.5 (Thermo Scientific) with the Sequest HT search engine. Analysis of MS spectra was performed using the following parameters: (1) trypsin as an enzyme with up to two missed cleavages; (2) precursor mass tolerance of 10 ppm; (3) fragment mass tolerance of 0.02 Da; (4) TMT of lysine and peptide N-terminus and carbamidomethylation of cysteine as fixed modifications; and (5) oxidation of methionine as variable modifications. Peptides were filtered at a false discovery rate (FDR) of 1% using the Percolator node. TMT quantification was performed using the Reporter Ions Quantifier node. Normalization was performed such that the total sum of the abundance values for each TMT channel over all peptides was the same.

Volcano plot analysis was performed using Perseus software version 2.1.4.0 (Tyanova et al., 2016), applying cutoff criteria of an absolute fold change (|FC|) greater than 2 and a *q* value <0.05, based on two-tailed Student's *t* tests with Benjamini–Hochberg FDR correction.

The MS proteomics data in this study has been deposited to the ProteomeXchange Consortium via the jPOST partner repository with the dataset identifiers PXD072266 (Fig. 4*a*,*b*) and PXD072265 (Fig. 4*c*,*d*).

### Electrophysiology

Six WT and *Nrxn1β* ΔHRD littermates of either sex at 4 weeks old were deeply anesthetized using a mix of intraperitoneally injected ketamine/xylazine (10 mg/ml ketamine, 1 mg/ml xylazine, in 0.9% saline; dose: 0.1 ml per 10 g of body weight) and decapitated. The brains were rapidly removed and placed in ice-cold slicing buffer containing the following (in mM): 2.5 KCl, 0.2 CaCl_2_, 4 MgCl_2_, 1.25 NaH_2_PO_4_, 26 NaHCO_3_, 10 D-glucose, and 25.2 sucrose bubbled with the pH equilibrated to 7.35 by 95% O_2_/5% CO_2_. Acute 350-µm-thick coronal slices containing the hippocampus were obtained using a VT1000S Vibratome (Leica Biosystems) and then incubated in carbogenated (5% CO_2_/95% O_2_) artificial cerebrospinal fluid (ACSF) containing the following (in mM): 125 NaCl, 2.5 KCl, 2 CaCl_2_, 1 MgCl_2_, 1.25 NaH_2_PO_4_, 26 NaHCO_3_, and 25 D-glucose at pH 7.35. Slices recovered in the incubation chamber at 32°C for 1 h, followed by incubation at room temperature before recordings. Subsequently, slices were transferred to a recording chamber and were continuously perfused with carbogenated ACSF for the duration of the experiments. Extracellular field excitatory postsynaptic potentials (fEPSPs) were evoked in the CA1 stratum radiatum by stimulating Schaffer collaterals with a concentric bipolar microelectrode (FHC) and recorded using a glass pipette filled with ACSF. fEPSPs were amplified (MultiClamp 700B amplifier, Molecular Devices) and digitized (Digidata-1550B interface, Molecular Devices) for analysis. Input/output (I/O) curves were obtained by plotting the fiber volley (FV) amplitudes against fEPSP slopes. The paired-pulse ratio was measured at varying interpulse intervals (25, 50, 100, 200, and 500 ms) and calculated as the slope of the second fEPSP divided by the slope of the first fEPSP. Extracellular recordings were acquired and digitized by Clampex 11.2, and analysis was done through Clampfit 11.2. Three to four slices per animal were recorded and averaged to generate one biological replicate (*n* = 6 means 6 animals). Statistical analysis was performed using two-way repeated-measures ANOVA for fEPSP versus Stimulation, FV amplitude versus Stimulation and PPR curves and simple linear regression slope comparison for the fEPSP versus FV amplitude curve. The results include the estimated difference between Nrxn1β ΔHRD and WT, the calculated *p* value, 95% confidence interval and partial *η*^2^ or *f*^2^, which represent effect size of the fixed effect (genotype).

### Behavior analysis

Mice were group-housed (two to five per cage) and maintained on a 12 h light/dark cycle (lights on 6:00–18:00). All behavioral experiments were carried out on age- and sex-matched WT and *Nrxn1β* ΔHRD mice (2–4 months old) during similar daytime periods in an isolated behavior room with the room lightning set as described in each test. Starting a week prior to behavioral testing, mice were handled by the experimenter over three 5 min sessions for habituation. Before every experiment, mice were transferred and habituated to the test room for at least 30 min. The apparatus was cleaned between subjects with a Peroxigard solution. The experimenter was blind to genotype for all experiments. In cohorts subjected to multiple behavioral assays, the tests were conducted in the following order: open field, Y-maze, elevated plus maze, rotarod, and contextual fear conditioning. Statistical analyses were performed using GraphPad Prism 10 and all the tests required either unpaired *t* tests or two-way repeated-measures ANOVA. The results include the estimated difference between *Nrxn1β* ΔHRD and WT, the *p* values, 95% confidence intervals, and partial *η*^2^ which represents the effect size due to the indicated factor in the case of two-way ANOVA, or simply the effect size *η*^2^ in the case of *t* tests.

#### Activity in metabolic cages

Mice were individually housed in cages from Promethion Core systems (Sable Systems International). This system uses infrared beams to monitor the activity of mice. Mice were placed in the cages for 7 d, and after a 3 d habituation period, the activity during the last 4 d was averaged to obtain the average daily activity (number of beam breaks per hour) for each mouse. Data was analyzed using ExpeData-P software (Sable Systems International).

#### Open field

The experimental setup included an open square field (L × W × H: 50 × 50 × 38 cm) under a 200 lux overhead light. Mice were placed in the center of the arena and allowed to freely explore for 10 min. Time spent in the center (25% of the area) and the periphery as well as total distance traveled was reported. Mouse behavior was video-recorded and analyzed using EthoVision XT17.5 software.

#### Y-maze

The Y-maze apparatus consists of three arms arranged at 120° angles from one another. A mouse is placed in the center of the maze and set free to explore the maze for 8 min. Spontaneous alternation behavior was assessed by recording the number of triads in which the mouse entered all three arms consecutively (i.e., an alternation) as well as the maximum number of possible alternations and the total distance traveled. Mouse behavior was video-recorded and analyzed using EthoVision XT17.5 software.

#### Elevated plus maze

The elevated plus maze consists of a chamber at a height of 90 cm above the floor in the shape of a plus (+) sign, with two open arms and two closed arms (surrounded by opaque walls). The maze was uniformly illuminated with overhead lighting at 200 lux. Each mouse was placed in the central square facing one of the closed arms and was allowed to freely explore the maze for 10 min. Mouse behavior was video-recorded and analyzed using EthoVision XT17.5 software.

#### Rotarod

Mice were trained during the first day on the rotarod apparatus (REF) in three trials (15 min intertrial interval). The speed of the rotarod started at 4 rpm and increased to 40 rpm over 300 s. The latency to fall from the rotarod was manually measured and plotted over the three trials to evaluate motor learning. On the second day, mice were placed on the rotarod with the same conditions and the averaged latency to fall from the rotarod across three trials was reported.

#### Fear conditioning

Fear conditioning was conducted using a standard operant box (Ugo Basile 46000 Fear Conditioning System) in two different contexts (A and B). Context A consisted of Plexiglass walls and a metal grid floor and was washed with 10% ethanol to provide a background odor. White noise at 55 dB provided an auditory background. Context B consisted of white and black striped walls and the metal grid floor was replaced by a uniform white Plexiglass floor. Vanilla scent was applied to substitute the ethanol background odor, and the white noise was turned off. On Day 1, mice were placed in context A for 2 min before fear conditioning using three pairings of the conditioned stimulus (CS duration 30 s, a tone of 85 dB, 2 kHz) with a coterminating unconditioned stimulus (US, 2 s electric footshock, 0.75 mA) at 1 min intervals. The mice remained in the training chamber for another 30 s before being returned to home cages. On Day 2, mice were placed in the same context A for 5 min without exposure to the CS or US to assess contextual memory. On Day 3, mice were placed in context B for 5 min before being exposed to the CS (1 min, 85 dB, 2 kHz) to assess auditory-cue memory. Mouse behavior was video-recorded and analyzed using EthoVision XT17.5 software. Motionless bouts lasting >1 s were considered freezing behavior.

### Statistical analysis

Statistical analyses were performed using GraphPad Prism (v10.2.3) or R (v4.5.0). Data distribution was assessed using built-in normality tests. When normality assumptions were not met, nonparametric tests were applied. A significance threshold of *α* = 0.05 was used for all analyses. Details regarding the specific statistical tests and data presentation are provided in the corresponding sections for each experiment and also available at https://doi.org/10.5061/dryad.k0p2ngfnk.

## Results

### In-frame deletion of the HRD from *Nrxn1β* does not alter its gene expression or the expression of other Nrxns

To investigate the in vivo physiological role of the Nrxn1β HRD, we used CRISPR-Cas9 genome editing to generate a mutant mouse line with in-frame deletion of the 93 bp encoding the HRD (Fig. 1*b*). Successful in-frame deletion was confirmed using PCR-based genotyping and subsequent sequencing (Fig. 1*c*, Extended Data Fig. 1-1). Homozygous *Nrxn1β ΔHRD* mice (*Nrxn1β* ΔHRD) showed no abnormalities at birth, and the offspring followed the expected Mendelian inheritance pattern (Fig. 1*d*), suggesting that there is no effect of Nrxn1β HRD deletion on embryonic development.

To assess expression of the mutant construct, we performed RT-qPCR analyses using hippocampal tissues from WT and *Nrxn1β* ΔHRD mice. Primer set 1 amplifies the HRD and was designed to confirm the Nrxn1β HRD deletion, whereas a primer set 2 was designed to assess the overall *Nrxn1β* mRNA expression (Fig. 1*e*, left). Primer set 1 failed to amplify anything in cDNA obtained from *Nrxn1β* ΔHRD mice (Fig. 1*e*, right), whereas primer set 2 showed comparable expression of *Nrxn1β* mRNA between the two genotypes. These results indicate that *Nrxn1β* ΔHRD mice express the truncated Nrxn1β at similar levels as full Nrxn1β is expressed in WT mice. By RT-qPCR, we further verified that deletion of the Nrxn1β HRD did not influence the expression levels of other Nrxns (Fig. 1*e*, right). Thus, the removal of the HRD from *Nrxn1β* has no significant impact on *Nrxn1β* gene expression or the expression of other Nrxns.

### *Nrxn1β* ΔHRD mice present with normal gross morphology and brain structure

We next performed anatomical characterization of 3-month-old *Nrxn1β* ΔHRD and WT mice. *Nrxn1β* ΔHRD mice of both sexes showed normal gross development (Fig. 2*a*,*b*), although the mutant males exhibited a modest, but statistically significant, reduction in body weight compared with WT controls. The size and gross morphology of the mutant brains seem normal relative to WT brains in both sexes (Fig. 2*c*,*d*). Histological analysis of sagittal brain cryosections stained with Cresyl violet further revealed no significant structural abnormalities in the hippocampus, cortex, or cerebellum in *Nrxn1β* ΔHRD mice (Fig. 2*e*,*f*). Together, these results suggest that *Nrxn1β* ΔHRD mice grow relatively normally and have anatomically normal-appearing brains.

### Deletion of the Nrxn1β HRD enhances Nlgn1 (B+)-mediated excitatory, but not inhibitory, presynaptic organization

**Figure 3.**
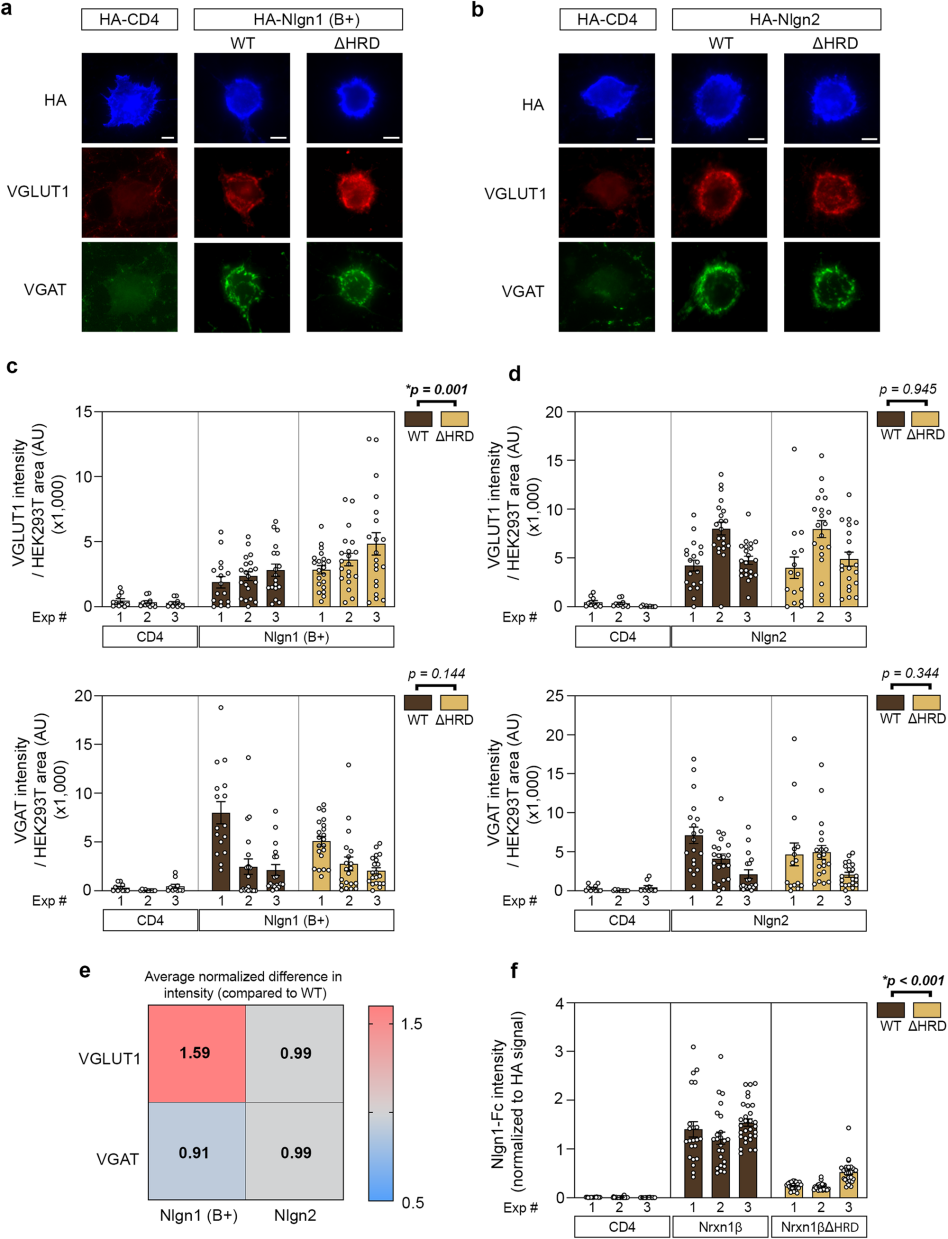
Evaluation of Nrxn1β- and Nrxn1β ΔHRD-mediated synaptogenic activity in primary cortical neurons. ***a***, Representative images of VGLUT1 and VGAT accumulation around HEK293T cells in artificial synapse formation assays. HEK293T cells were transfected with either HA-Nlgn1 (B+), a β-Nrxn-specific ligand, or HA-CD4 as a negative control and cocultured with Nrxn1β WT or ΔHRD-expressing neurons. Cells were stained with anti-HA (blue), anti-VGLUT1 (red), or anti-VGAT (green). Scale bar, 5 µm. ***b***, Quantification of VGLUT1 and VGAT intensity around HA-Nlgn1 (B+) or HA-CD4 transfected HEK293T cells. Statistical significance was assessed using a linear mixed-effects model. Data are presented as mean ± SEM. *n* = 3 independent experiments (at least 10 images per condition in each experiment were analyzed). HA intensity for each condition is presented in Extended Data Figure 3-1*a*. ***c***, Representative images of VGLUT1 and VGAT accumulation around HEK293T cells in an artificial synapse formation assay. HEK293T cells were transfected with either HA-Nlgn2, a pan-Nrxn ligand, or HA-CD4 as a negative control. Cells were stained with anti-HA (blue), anti-VGLUT1 (red), or anti-VGAT (green). Scale bar, 5 µm. ***d***, Quantification of VGLUT1 and VGAT intensity around HA-Nlgn2 or HA-CD4 transfected HEK293T cells. Statistical significance was assessed using a linear mixed-effects model. Data are presented as mean ± SEM. *n* = 3 independent experiments (at least 9 images per condition in each experiment were analyzed). HA intensity for each condition is presented in Extended Data Figure 3-1*b*. ***e***, Heatmap presenting the average normalized difference in intensity relative to WT across all of the previously described conditions. Values above 1 indicate an increase in intensity while values below 1 indicate a decrease. ***f***, Cell surface binding assay in COS7 cells transfected with HA-CD4, HA-Nrxn1β, or HA-Nrxn1βΔHRD measuring the binding affinity to Nlgn1 (B+)-Fc. The graph shows the Nlgn1 (B+)-Fc average intensity normalized to the HA intensity of the same cell for each condition over three independent experiments. Statistical significance was assessed using a linear mixed-effects model. Data are presented as mean ± SEM. *n* ≥ 22 analyzed cells for each experiment. HA intensity for each condition is presented in Extended Data Figure 3-1*c*. Assessments of excitatory and inhibitory in vitro synapse density are presented in Extended Data Figure 3-2*a–d*.

To investigate the functional role of the HRD in synaptic organization, we performed artificial synapse formation assays, which test the ability of molecules to influence clustering of pre- and postsynaptic proteins (Scheiffele et al., 2000). HEK293T cells were transfected with either the β-Nrxn-specific ligand Nlgn1 harboring an insert at splicing site B (Nlgn1 B+), or Nlgn2, which does not possess this splicing site and binds all Nrxn isoforms. These transfected cells were then cocultured with primary cortical neurons prepared from WT or *Nrxn1β* ΔHRD mice, and presynaptic organization was assessed by quantifying the accumulation of the presynaptic markers VGLUT1 and VGAT on HEK293T cells (Fig. 3*a*,*b*). Notably, deletion of the HRD led to a statistically significant increase in VGLUT1, but not VGAT, accumulation at hemi-synapses induced by Nlgn1 (B+)-expressing HEK293T cells (Fig. 3*c*). This result was not due to a change in Nlgn1 (B+) expression as it was similar in all conditions (Extended Data Fig. 3-1*a*). These results suggest a modulatory role of the Nrxn1β HRD that is specific to excitatory presynaptic assembly. In contrast, we observed no differences in VGLUT1 or VGAT clustering in neurons cocultured with Nlgn2-expressing HEK293T cells (Fig. 3*d*, Extended Data Fig. 3-1*b*), indicating that HRD deletion does not affect presynaptic organization mediated by pan-Nrxn ligands but seems to be confined to that mediated by the β-Nrxn-specific ligand Nlgn1 (B+; summarized in Fig. 3*e*).

10.1523/ENEURO.0399-25.2026.f3-1 Figure 3-1 **Quantification of surface expression levels of the extracellular HA-tagged constructs** **(a – b)** Quantification of the average intensity of surface HA on HEK293 T cells expressing either HA-Nlgn1 (B+) or HA-Nlgn2 used in Fig. 3. Statistical significance was assessed using a linear mixed-effects model. Data are presented as mean ± SEM. n ≥ 9 cells for each experiment. **(c)** Quantification of surface expression intensity of HA-tagged CD4, Nrxn1β and Nrxn1βΔHRD. Nrxn1βΔHRD surface expression appears slightly higher than that of the WT protein with an estimated increase of 2570 units (95% CI: [227–4914]), which is small compared to the within-experiment variability. Therefore, we consider the expression level comparable. Statistical significance was assessed using a linear mixed-effects model. Data are presented as mean ± SEM. n ≥ 22 cells for each experiment. Download Figure 3-1, TIF file.

10.1523/ENEURO.0399-25.2026.f3-2 Figure 3-2 **Comparison of excitatory and inhibitory synapse density in primary cortical neurons from Nrxn1β ΔHRD and WT mice** **(a)** Representative images of Nrxn1β WT or ΔHRD-expressing neurons immunostained for VGLUT1 (green) and PSD95 (red) to mark excitatory pre- and post-synaptic compartments, respectively. MAP2 (blue) is used in all conditions as a marker of dendrites. Scale bar: 2 µm. **(b)** Quantification of VGLUT1-positive PSD95 puncta (representative of excitatory synapses) per micrometer on secondary dendrites. Statistical significance was assessed using a linear mixed-effects model. Data are presented as mean ± SEM. n = 3 independent experiments (at least 7 images per experiment were analyzed). **(c)** Representative images of Nrxn1β WT or ΔHRD-expressing neurons immunostained for VGAT (green) and gephyrin (red) to mark inhibitory pre- and post-synaptic compartments, respectively. MAP2 (blue) is used in all conditions as a marker of dendrites. Scale bar: 2 µm. **(d)** Quantification of VGAT-positive gephyrin puncta (representative of inhibitory synapses) per micrometer on secondary dendrites. Statistical significance was assessed using a linear mixed-effects model. Data are presented as mean ± SEM. n = 3 independent experiments (at least 7 images per experiment were analyzed). Download Figure 3-2, TIF file.

To explore the mechanism underlying the increased VGLUT1 accumulation observed upon HRD deletion, we assessed whether the affinity between Nlgn1 (B+) and Nrxn1β is altered in the absence of the HRD. A higher binding affinity could potentially stabilize the trans-synaptic complex and account for the enhanced excitatory presynaptic recruitment. To test this, we transfected COS7 cells with either extracellularly HA-tagged CD4, HA-Nrxn1β full-length, or HA-Nrxn1β ΔHRD constructs and quantified surface binding of Nlgn1 (B+)-Fc by immunocytochemistry. Contrary to our initial expectation, deletion of the HRD resulted in a ∼75% reduction in the binding of Nlgn1 (B+; Fig. 3*f*) even though surface expression of all HA-tagged constructs was comparable (Extended Data Fig. 3-1*c*). These results suggest that, although HRD deletion diminishes the binding affinity of Nrxn1β for Nlgn1 (B+), some compensatory mechanisms occur and potentially support the enhanced excitatory synapse maturation.

Following the observation that VGLUT1 accumulation was strengthened in cells expressing Nrxn1β ΔHRD, we next wondered whether HRD deletion affected synaptic density. Primary cortical neurons derived from WT or *Nrxn1β ΔHRD* mice were immunostained for the dendritic marker MAP2 alongside excitatory synapse markers VGLUT1 and PSD95 and inhibitory markers VGAT and gephyrin (Extended Data Fig. 3-2*a*, 3-2*c*). Quantitative analysis of synapse number revealed no significant difference in synaptic density between WT and ΔHRD-expressing neurons (Extended Data Fig. 3-2*b*, 3-2*d*), indicating that the HRD domain is dispensable for synapse formation and maintenance in vitro.

### Synaptosomal protein expression is unchanged in both the cortex and the hippocampus of *Nrxn1β* ΔHRD mice

**Figure 4.**
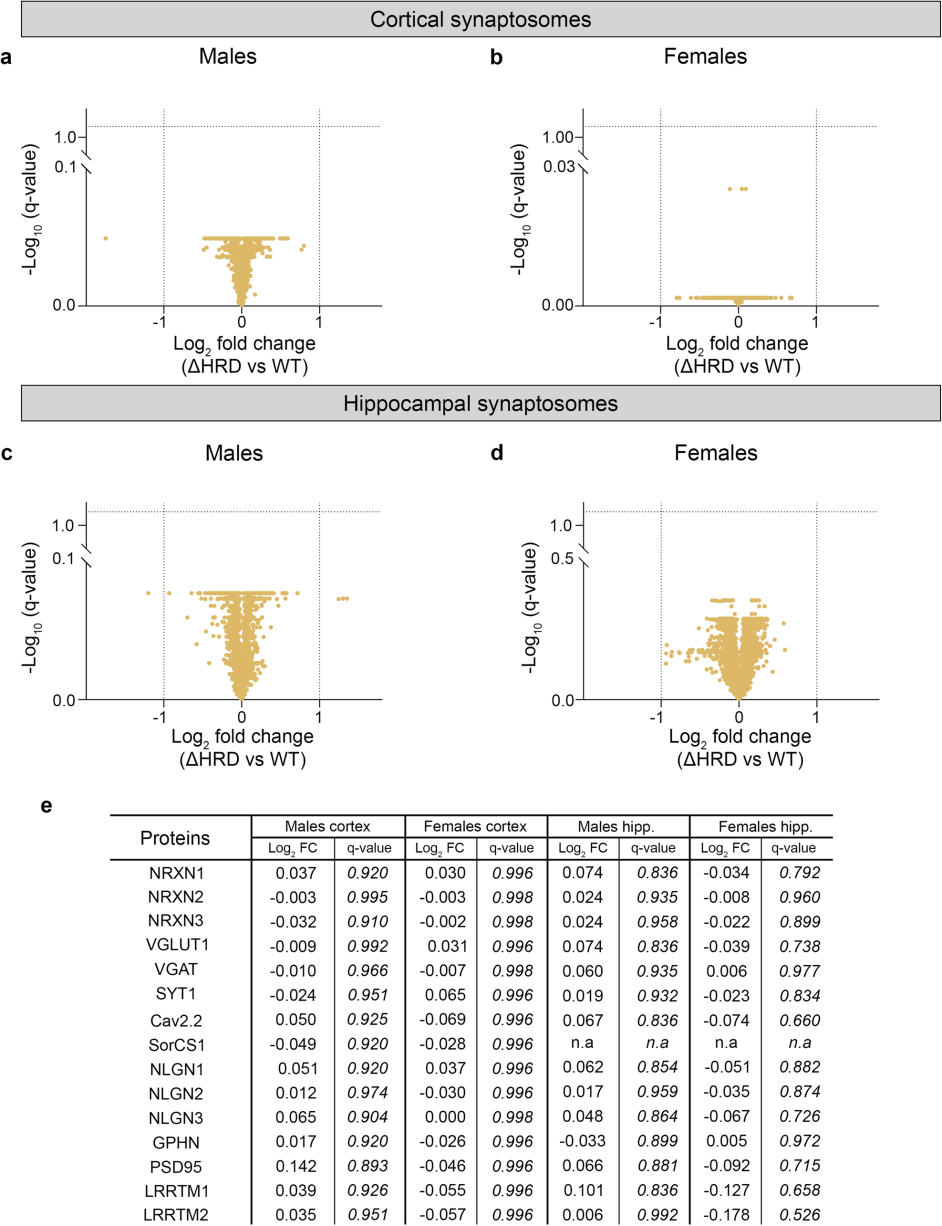
Proteomic analysis of cortical and hippocampal synaptosomes from *Nrxn1β* WT and *Nrxn1β* ΔHRD mice. ***a–d***, Volcano plots showing the fold change in expression of synaptic proteins in the cortex (top panel) or hippocampus (bottom panel) in male (***a***, ***c***) and female (***b***, ***d***) *Nrxn1β* ΔHRD mice compared with WT mice identified by LC-MS/MS. The dashed lines represent the cutoffs for significant changes and have been fixed to −1 and 1 for the log_2_ fold change axis and 1.3 for the -log_10_ (*q* value) axis which represents a *q* value of 0.05. ***e***, Table listing several pre- and postsynaptic proteins detected during the proteomic analysis showing no difference in expression compared with WT.

Nrxns primarily perform their functions at the presynaptic membrane, where they facilitate the connection between pre- and postsynaptic compartments through interactions with multiple postsynaptic partners, playing a critical role in neurotransmission. We therefore next assessed the impact of Nrxn1β HRD deletion on synaptic molecular integrity using quantitative proteomic analysis of cortical and hippocampal synaptosomes from WT and *Nrxn1β* ΔHRD mice. In both the cortex and the hippocampus, no significant differences in the abundance of any synaptic proteins were detected in either male or female *Nrxn1β* ΔHRD mice compared with WT controls (Fig. 4*a–d*). A representative subset of the detected proteins is listed in Figure 4*e*. Notably, none of the three Nrxn isoforms or Nrxn ligands such as Nlgn1/2/3 or LRRTM2 exhibited changes in abundance in the absence of the HRD. Similarly, key pre- and postsynaptic proteins such as synaptotagmin 1 (SYT1), VGLUT1, and PSD95 showed no significant alterations in expression level. Of note, although previous findings have demonstrated that SorCS1 interacts with Nrxn1β via the HRD (Lee et al., 2023a), synaptosomal expression of SorCS1 was unaffected by loss of the HRD, at least in the cortical tissues where it was detectable. Together, these data suggest that deletion of the Nrxn1β HRD has no effect on synaptic molecular integrity.

### Deletion of the Nrxn1β HRD alters presynaptic short-term plasticity in excitatory synapses

**Figure 5.**
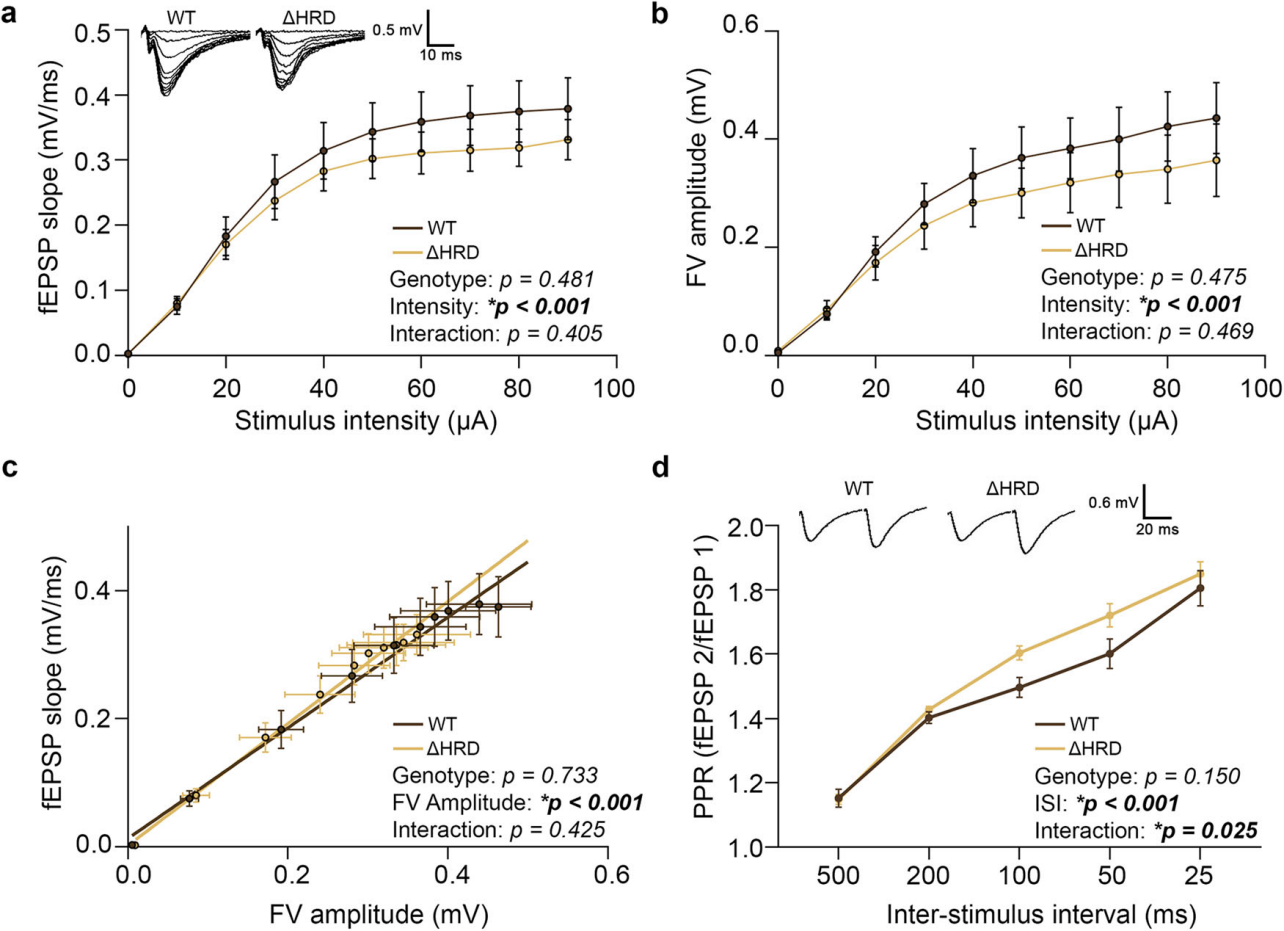
Assessment of excitatory basal neurotransmission and presynaptic short-term plasticity in *Nrxn1β* ΔHRD mice through fEPSP recordings. ***a***, Input/output (I/O) curves presenting the postsynaptic response (fEPSP slope) to increasing stimulation of the Schaffer collateral fibers in the hippocampus. Statistical significance was examined by two-way repeated-measures ANOVA. Data are presented as mean ± SEM. *n* = 6/6 mice (WT/ΔHRD; one point represents averaged results from 6 mice per genotype with recordings from 3–4 slices per mouse). ***b***, Fiber volley amplitudes related to stimulation intensity. Statistical significance was examined by two-way ANOVA. Data are presented as mean ± SEM. *n* = 6/6 mice (WT/ΔHRD; one point represents averaged results from 6 mice per genotype with recordings from 3–4 slices per mouse). ***c***, Linear regression curve of fEPSP slope related to fiber volley amplitude. Statistical significance was examined by using a linear regression model. Data are presented as mean ± SEM. *n* = 6/6 mice (WT/ΔHRD; one point represents averaged results from 6 mice per genotype with recordings from 3–4 slices per mouse). ***d***, Evolution of the paired-pulse ratio with decreasing interstimulus intervals. Statistical significance was examined by two-way repeated-measures ANOVA. Data are presented as mean ± SEM. *n* = 6/6 mice (WT/ΔHRD; one point represents averaged results from 6 mice per genotype with recordings from 3–4 slices per mouse).

Since presynaptic organization at excitatory synapses seems altered by HRD deletion while the formation, maintenance, and molecular integrity of synapses remain normal, we next investigated synaptic function. Using acute hippocampal slices from *Nrxn1β* ΔHRD or WT littermates, we recorded field excitatory postsynaptic potentials (fEPSPs) at Schaffer collateral (SC)→CA1 synapses. To assess basal synaptic transmission, input/output (I/O) curves were generated to examine how fEPSP slope and fiber volley (FV) amplitude varied with increasing stimulation intensity, as well as their relationship (Fig. 5*a–c*). While both the fEPSP and FV curves exhibited a nonsignificant downward shift in *Nrxn1β* ΔHRD mice, the correlation between fEPSP slope and FV amplitude was nearly identical across genotypes. This suggests that the postsynaptic response increases proportionally with the number of recruited fibers upon stimulation in both groups in a comparable manner, indicating no major change in basal evoked synaptic transmission.

At the same synapses, we also assessed the paired-pulse ratio (PPR), a measure of short-term presynaptic plasticity that reflects changes in synaptic strength following two closely spaced stimuli (Regehr, 2012). According to the literature, stimulation of SC→CA1 synapses typically results in facilitation (PPR > 1), which was also observed in both WT and *Nrxn1β* ΔHRD mice. While PPR values were not statistically different at individual interstimulus intervals (ISIs), they appeared raised in *Nrxn1β* ΔHRD mice at shorter intervals (50 and 100 ms). This trend was supported by a statistically significant genotype × ISI interaction (*p* = 0.025), suggesting that ΔHRD mice may respond differently to varying ISIs (Fig. 5*d*). Such paired-pulse facilitation (PPF) is often interpreted as an indirect readout of synaptic vesicle release probability (Pr), where a lower initial Pr results in higher facilitation. Increased PPR in *Nrxn1β* ΔHRD mice might therefore reflect a decrease in Pr. Together, these results suggest that the Nrxn1β HRD may be involved in modulating presynaptic short-term plasticity without affecting basal synaptic transmission at SC→CA1 synapses.

### No locomotion deficits or anxiety behavior were detected in *Nrxn1β* ΔHRD mice

**Figure 6.**
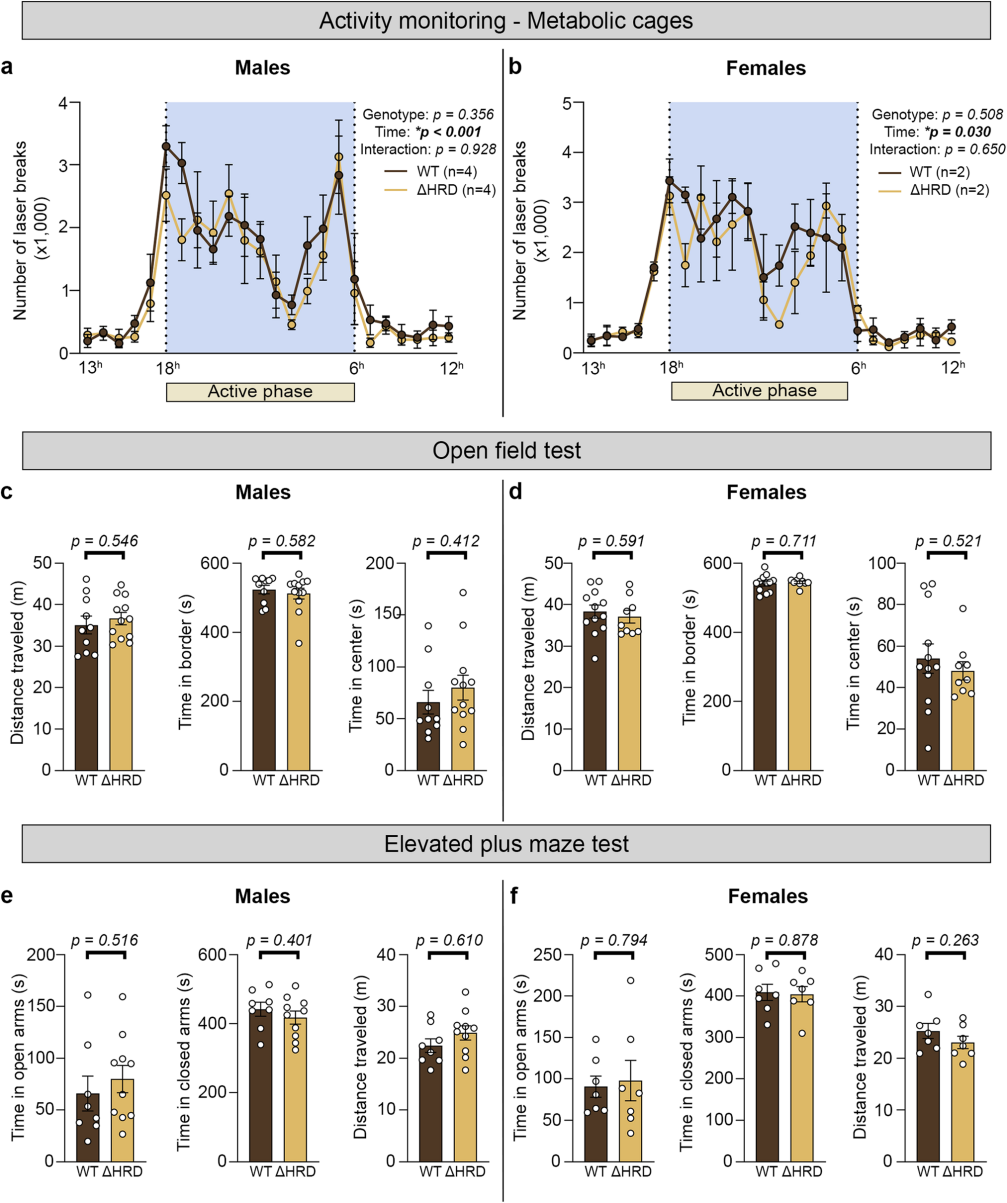
Evaluation of basic locomotor functions and anxiety-like behavior in Nrxn1β ΔHRD mice. ***a***, ***b***, Average daily basal locomotor activity in *Nrxn1β* WT and ΔHRD males (***e***) and females (***f***) evaluated over a 4 d period in metabolic cages. Statistical significance was examined by two-way repeated-measures ANOVA. Data are presented as mean ± SEM. *n* = 4/4 males (WT/ΔHRD) and 2/2 females (WT/ΔHRD). ***c***, ***d***, Open field test in *Nrxn1β* WT and ΔHRD males (***a***) and females (***b***). Left graphs, Time spent in the anxiogenic center area of the open field box. Middle graphs, Time spent in the safe area (border) of the open field box. Right graphs, Total distance traveled during the test. Statistical significance was examined by unpaired *t* tests. Data are presented as mean ± SEM. *n* = 10/12 males (WT/ΔHRD) and 12/9 females (WT/ΔHRD). ***e***, ***f***, Time spent in open (left graphs) and closed arms (middle graphs) of the EPM by male (***c***) or female (***d***) *Nrxn1β* WT and *Nrxn1β* ΔHRD mice. Total distance traveled is also presented for males and females (right graphs). Statistical significance was examined by unpaired *t* tests. Data are presented as mean ± SEM. *n* = 8/10 males (WT/ΔHRD) and 7/7 females (WT/ΔHRD).

We next assessed the consequences of Nrxn1β HRD deletion on mouse behavior. Basal locomotor activity was primarily investigated through two experiments: (1) 4 d observation in cages with an infrared beam-break system and (2) 10 min free exploration in an open field (OF). Preliminary assessment of circadian activity locomotion patterns suggests no difference between genotypes in either males or females (Fig. 6*a*,*b*). Additionally, total traveled distance in OF testing was also similar (Fig. 6*c*,*d*), suggesting that Nrxn1β HRD deletion does not affect basal locomotor activity. Next, anxiety-like behavior was evaluated using OF and elevated plus maze (EPM) tests, which leverage mice aversion to open spaces and their innate exploratory drive. In the OF test, *Nrxn1β* ΔHRD mice of both sexes spent comparable amounts of time as WT controls in the anxiogenic center and safer peripheral zones (Fig. 6*c*,*d*). In the EPM test, *Nrxn1β* ΔHRD mice exhibited similar open arm exploration behavior as WT mice regardless of sex (Fig. 6*e*,*f*). Collectively, these data indicate that deletion of the Nrxn1β HRD does not lead to basal locomotor dysfunction or anxiety-like behaviors.

### Learning and memory appear intact in *Nrxn1β* ΔHRD mice

**Figure 7.**
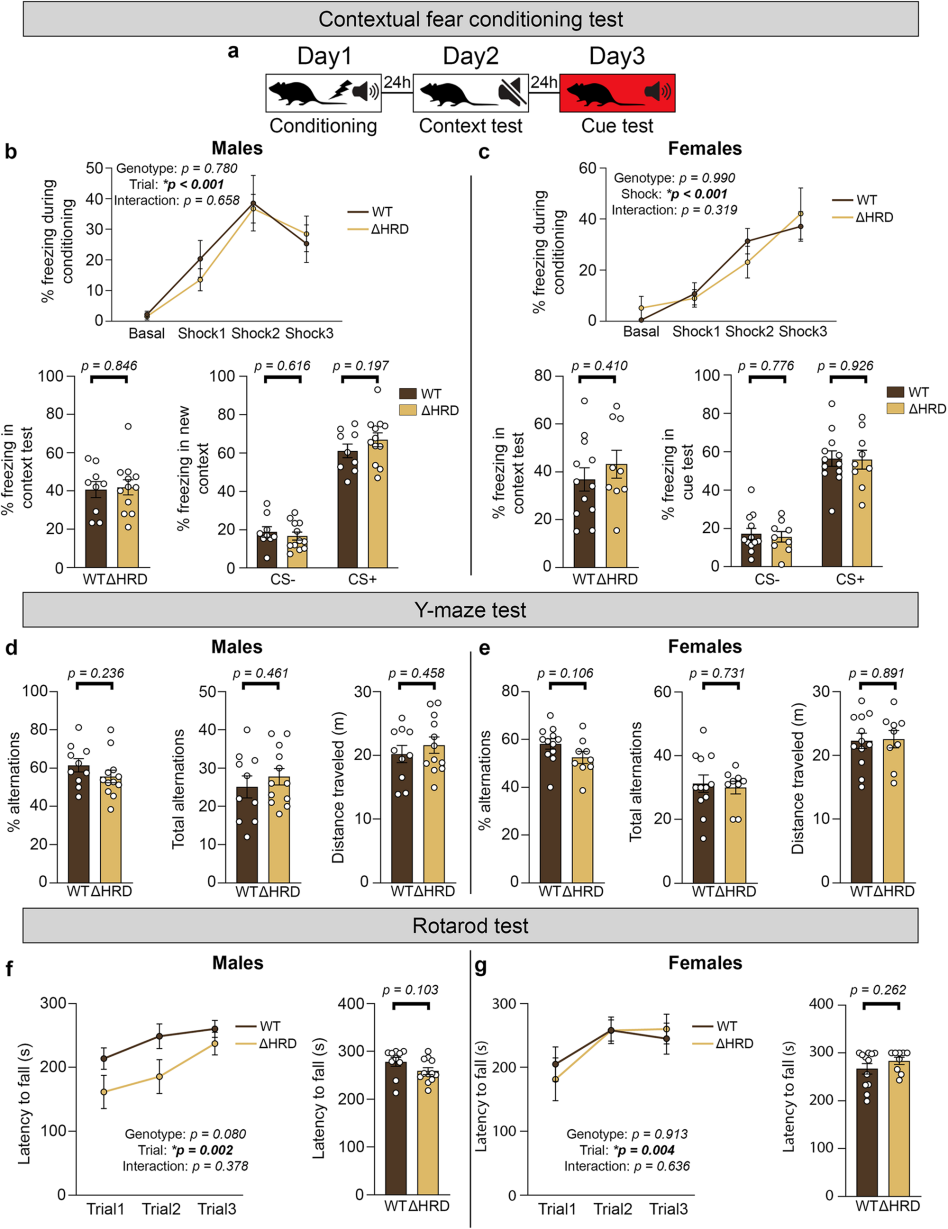
Learning and memory abilities of *Nrxn1β* ΔHRD mice compared with WT mice. ***a***, Graphical representation of the fear conditioning test. During Day 1, mice are presented with the paired conditioning (tone) and unconditioned (shocks) stimuli three times. On Day 2, mice are re-exposed to the same context without any presentation of the stimuli. On Day 3, mice are placed in a different context without any stimuli first and are then presented with the conditioning stimulus. ***b***, ***c***, Percentage of freezing behavior observed in *Nrxn1β* WT and ΔHRD male (left panel) or female (right panel) mice during the different steps of the contextual fear conditioning test. Top graphs, Percentage of freezing behavior after presentation of the three unconditioned stimuli (shocks) during the conditioning phase. Bottom left graphs, Percentage of freezing behavior during the context test. Bottom right graphs, Percentage of freezing behavior during the cue test before and after presentation of the conditioning stimulus (CS−, CS+, respectively). Statistical significance was examined by two-way repeated-measures ANOVA (top graphs), unpaired *t* tests (bottom left graphs), and two-way repeated-measures ANOVA followed by Fisher's LSD test for multiple comparisons (bottom right graphs). Data are presented as mean ± SEM. *n* = 9/12 males (WT/ΔHRD) and 12/9 females (WT/ΔHRD). ***d***, ***e***, Percentage alternation (left graphs), total alternations (middle graphs), and total distance traveled (right graphs) of male (***c***) and female (***d***) *Nrxn1β* WT and ΔHRD mice during Y-maze testing. Statistical significance was examined by unpaired *t* tests. Data are presented as mean ± SEM. *n* = 10/12 males (WT/ΔHRD) and 12/9 females (WT/ΔHRD). ***f***, ***g***, Latency to fall from the rotarod during each trial of the training day (left graphs) and average latency to fall from the rotarod over three trials during the test day (right graphs) in both male (***f***) and female (***g***) *Nrxn1β* WT and ΔHRD mice. Statistical significance was examined by two-way repeated-measures ANOVA (left graphs) and unpaired *t* tests (right graphs). Data are presented as mean ± SEM. *n* = 10/12 males (WT/ΔHRD) and 12/9 females (WT/ΔHRD).

Given the high expression of Nrxn1β in the hippocampus and cortex (Uchigashima et al., 2019), we next assessed learning and memory in *Nrxn1β* ΔHRD mice. A previous study showed that deletion of all β-Nrxns [Nrxn1/2/3β knock-out (KO)] in the hippocampal CA1 is associated with impaired contextual fear memory (Anderson et al., 2015). We therefore evaluated associative learning and memory using the same contextual fear conditioning paradigm as in this previous study. Twenty-four hours after conditioning with paired conditioned (tone) and unconditioned (footshock) stimuli in a particular context, freezing behavior in response to re-exposure to this original context was measured. Forty-eight hours later, mice were exposed to a novel context both without and with the conditioned stimulus to evaluate fear memory discrimination and cued fear memory, respectively (Fig. 7*a*). Both male and female *Nrxn1β* ΔHRD mice exhibited similar freezing levels as compared with WT controls during the conditioning phase (Fig. 7*b*,*c*, top) and comparable fear learning. *Nrxn1β* ΔHRD and WT mice re-exposed to the same context after 24 h showed equivalent freezing behavior, regardless of sex (Fig. 7*b*,*c*, bottom left), suggesting intact contextual fear memory. When placed in a different context without presentation of any stimulus, both genotypes froze less frequently (Fig. 7*b*,*c*, bottom right) indicating preserved context discrimination. Finally, after presentation of the tone, both genotypes showed similar freezing responses, revealing no genotype-linked difference in cued memory.

Next, spatial working memory was tested using the Y-maze test, which evaluates the natural tendency of rodents to explore novel arms. Both the percentage of alternations and the maximum possible alternations were comparable between genotypes in males and females (Fig. 7*c*,*d*), suggesting that short-term spatial working memory is intact in *Nrxn1β* ΔHRD mice.

We then evaluated motor learning and coordination using a rotarod test. We observed no statistically significant differences in latency to fall between *Nrxn1β* ΔHRD and WT mice of either sex during the learning phase (Fig. 7*a*,*b*, left), although in males, the near-significant *p* value (mean genotype difference: −46.2 s, 95% CI [−98.41, 6.01], *p* = 0.080) and the large effect size (*η*^2^ = 0.270) might indicate a possible trend toward a reduced latency to fall in *Nrxn1β* ΔHRD male mice. However, overall motor learning was comparable between genotypes, as demonstrated by the similar progression profile throughout the trials. During the test phase, no difference between genotypes was observed in the average latency to fall from the rotarod across three trials (Fig. 7*f*,*g*, right). Despite not being statistically significant, the difference between male WT and *Nrxn1β* ΔHRD mice (mean difference: −19.50 s, 95% CI [−43.32, 4.332], *p* = 0.103) supports the observation made during the learning phase. Nonetheless, these results indicate that deletion of the Nrxn1β HRD does not significantly alter motor learning or coordination in mice.

## Discussion

Just as the emergence of three independent Nrxn genes in vertebrates suggests diversification of their functions, the appearance of a distinct promoter producing the shorter β-isoform raises questions about its functional redundancy and divergence relative to the ancestral α-Nrxns. We thus generated and characterized a novel genetically modified mouse line bearing a truncated form of Nrxn1β lacking the HRD, the only region not also found in α-Nrxns. Our results show that this deletion leads to impaired presynaptic short-term plasticity as well as abnormally facilitated Nlgn1 (B+)-induced excitatory presynaptic differentiation.

Morphological characterization revealed that *Nrxn1β* ΔHRD mice grow normally and are viable, although a modest 5% reduction in body weight was observed in young adult *Nrxn1β* ΔHRD males. A previous study of *β-Nrxn* KO mice showed that mice lacking Nrxn2β or Nrxn3β showed significant body weight reduction, especially in adults (>3 months old) whereas *Nrxn1β* KO produced no effect on mouse body weight over time (Anderson et al., 2015). Thus, there are conflicting effects of Nrxn KO on body weight. In addition, as weight reduction is frequently reported in KO models (Reed et al., 2008), the subtle body weight effects observed in this study could simply be an off-target effect of the genetic manipulation procedure and unrelated to the targeted gene. Aside from general growth assessed by measuring body weight, no gross abnormalities were detected in the brains of young adult *Nrxn1β* ΔHRD mice, suggesting that the HRD of Nrxn1β is dispensable for overall structural brain development and prompted a search for effects of the deletion at the cellular and synaptic level.

Nrxns are well known for having multiple binding partners with interactions often governed by alternative splicing (Sudhof, 2017). Among these partners, neuroligins (Nlgns) are the most extensively studied (Ichtchenko et al., 1995). Notably, Nlgn1 contains an alternative splicing site (B) that selectively allows for interaction with β-Nrxns when the insert is present (Nlgn1 B+; Boucard et al., 2005; Craig and Kang, 2007). The fact that the most abundant Nlgn1 isoform in mouse cortex, hippocampus, and cerebellum includes this insertion suggests that β-Nrxn/Nlgn1 signaling is crucial for establishing proper synaptic properties, a notion supported by autism-associated mutations found in both Nrxn1β and Nlgn1 (Feng et al., 2006; Camacho-Garcia et al., 2012; Camacho-Garcia et al., 2013; Nakanishi et al., 2017; Lee et al., 2023b). We therefore focused on this interaction in our artificial synapse formation assays. These showed a ∼50% increase in the Nlgn1 (B+)-induced accumulation of the excitatory presynaptic marker VGLUT1, but not the inhibitory marker VGAT, in Nrxn1β ΔHRD neurons, pointing toward a specific alteration in excitatory presynaptic organization.

Several mechanisms or a combination thereof might explain these results: (1) deletion of the HRD could increase Nrxn1β affinity for Nlgn1, thus stabilizing the trans-synaptic complex; (2) HRD deletion could modify Nrxn1β trafficking, clustering, and/or membrane turnover; (3) since Nrxn1β seems to recruit synaptic vesicles through interaction with yet uncharacterized presynaptic coreceptors (Gokce and Sudhof, 2013; Rabaneda et al., 2014), HRD deletion might enhance activity of an excitatory-specific coreceptor. Our immunocytochemical data do not support the first potential explanation as we observed a ∼75% decrease in Nrxn1β–Nlgn1 (B+) binding when the HRD was removed. It is important to note that this reduction might be overestimated in our non-neuronal system as Nrxn–Nlgn interactions in the central nervous system can also be modulated by post-translational modifications that would not be replicated in our assay (e.g., tissue-specific heparan sulfate modification; Zhang et al., 2018). Regarding the second possible mechanism, previous work has identified sortilin-related VPS10 domain containing receptors 1 and 2 (SorCS1 and SorCS2) as Nrxn1β ligands, our group has confirmed that their surface interaction is HRD dependent (Lee et al., 2023a), and SorCS1 can regulate axonal surface trafficking of Nrxn1β (Savas et al., 2015; Ribeiro et al., 2019; Lee et al., 2023a). More generally, SorCS proteins regulate protein trafficking and receptor recycling and act as endocytic receptors upon ligand binding (Hermey, 2009). Deletion of the Nrxn1β HRD might therefore compromise the interaction between β-Nrxns and SorCS1, thereby abnormally stabilizing β-Nrxn surface expression and enhancing its downstream presynaptic signaling, thus leading to the observed VGLUT1 overaccumulation. The third mechanism also remains plausible as an increased presence of Nrxn1β ΔHRD at the surface could overactivate a coreceptor. While these mechanisms require further investigation, our results highlight the potential importance of HRD-dependent dynamics of Nrxn1β in regulating excitatory presynaptic organization.

Field recordings in the hippocampal CA1 region upon SC stimulation revealed an alteration in presynaptic short-term plasticity as characterized by increased PPR at varying ISIs, suggesting the involvement of Nrxn1β HRD in Pr modulation. Such modifications are commonly attributed to changes in presynaptic calcium dynamics (Citri and Malenka, 2008; Selten et al., 2016; Jackman and Regehr, 2017). Of note, while partially similar, as the synaptic changes were particularly observable at 50 and 100 ms ISIs, our PPR phenotype seems to indicate an additional or alternative mechanism involving vesicle dynamics. Indeed, similar PPR patterns have been found in studies looking at dysfunction of the presynaptic protein Rab3A, which is a synaptic vesicle-associated GTP-binding protein that facilitates vesicle priming (Sakane et al., 2006; Schluter et al., 2006). Moreover, deletion of Nlgn1 in cultured neurons has been shown to affect maturation and the number of recycling vesicles (Wittenmayer et al., 2009), also supporting an effect on vesicle dynamics due to reduced Nrxn1β–Nlgn1 binding. Regarding presynaptic calcium dynamics, a previous study by Anderson et al. implicated β-Nrxns in tonic regulation of short-term plasticity through modulation of the endocannabinoid pathway. Specifically, they showed that presynaptic β-Nrxns regulate postsynaptic 2-AG synthesis, which retrogradely modulates presynaptic endocannabinoid receptor 1 (CB1R) activity (Anderson et al., 2015). CB1R is known to influence both short- and long-term synaptic plasticity in the hippocampus, in part by modulating voltage-gated calcium channel activity (Wilson and Nicoll, 2001; Wilson et al., 2001; Nemeth et al., 2008). Based on these findings, β-Nrxns are proposed to regulate calcium influx by influencing basal presynaptic CB1R tone and therefore glutamate Pr. In this model, HRD deletion would suppress the inhibitory effect of Nrxn1β on the synthesis of postsynaptic 2AG, thereby increasing the activity of presynaptic CB1R and diminishing Ca^2+^ influx, resulting in an overall decrease in Pr. Taken together, while PPR traces are reminiscent of an altered vesicle dynamics, additional contributions from presynaptic calcium influx modulation through CB1R cannot be excluded and require further investigation.

Despite the functional impairments observed in our electrophysiology experiments, no behavioral deficits were observed in either *Nrxn1β* ΔHRD males or females across the basic cognitive tests performed in this study, in contrast with what has been observed in the triple β-Nrxn KO model (Anderson et al., 2015). This discrepancy may reflect functional compensation by other β-Nrxns, which also harbor homologous HRD sequences. It is noteworthy that preliminary testing of locomotor functions did not reveal basal impairments, although a trend toward fine motor dysfunctions was observed in males and may require further investigation using more specialized tests. To fully elucidate the functional contribution of the HRD domain across the neurexin family, future models lacking the HRD in all three β-Nrxns might be necessary to uncover the full functions of this domain.

Finally, as mentioned in the introduction, the HRD of β-Nrxns interacts with several proteins involved in neuropathologies such as amyloid-β oligomers and α-synuclein preformed fibrils relevant to Alzheimer's disease and Parkinson's disease, respectively. Notably, the HRD displays much higher affinity for the aggregated, pathological forms than for their physiological counterparts, indicating that specific biochemical features of the HRD may mediate this selective interaction. More comprehensive studies of these properties are needed to elucidate the mechanism behind this specific interaction and potentially act as a basis for the development of therapeutic compounds targeting responses to the aggregates. In this context, mouse lines such as the one presented here represent a useful tool to further characterize these interactions in vivo.

## Synthesis

Reviewing Editor: Eran Perlson, Tel Aviv University Decisions are customarily a result of the Reviewing Editor and the peer reviewers coming together and discussing their recommendations until a consensus is reached. When revisions are invited, a fact-based synthesis statement explaining their decision and outlining what is needed to prepare a revision will be listed below. The following reviewer(s) agreed to reveal their identity: Shuwen Chang, Emmanuel Prikas. Note: If this manuscript was transferred from JNeurosci and a decision was made to accept the manuscript without peer review, a synthesis may not be available.

Overall, this is a well-executed and clearly written study defining a domain-specific role for the histidine-rich region (HRD) of Neurexin1β in regulating synapse formation, short-term plasticity, and downstream behavioral effects, and it represents a meaningful conceptual advance by decoupling binding affinity from synaptogenic potency and revealing the counter-intuitive enhancement of VGLUT1 accumulation despite reduced Nlgn1-Nrxn1β affinity.

Before publication, however, several issues require clarification and revision. (1) The behavioral dataset in Fig. 6a,b suffers from insufficient sample size, particularly for females (n=2), preventing valid inference on sex-dependent effects; the authors should either increase the number of animals (ideally to {greater than or equal to}4 per genotype and sex) or temper their conclusions and clearly frame these data as preliminary trends. (2) If the authors wish to retain the contextual fear conditioning (CFC) results, the experiment must be reperformed with a properly matched testing environment, including both the contextual and auditory cues used during conditioning; otherwise, the current CFC section should be removed. (3) Potential pseudoreplication must be addressed in Fig. 5 by explicitly stating whether the n reflects the number of mice or the number of slices used in the repeated-measures ANOVA. (4) Several methodological descriptions require clarification, including the trans-synapse formation assay (briefly explaining the neuron-HEK co-culture and VGLUT1/VGAT readouts) and the binding-affinity experiment (explicitly describing the use and purpose of CD4 constructs). (5) The proteomics volcano plot (Fig. 4) would benefit from an adjusted y-axis to improve visualization of differentially expressed proteins. (6) The statistical analysis for the rotarod assay should be rechecked, as there appears to be a trend in HRD males that may have been overlooked. (7)The discussion should revise claims regarding CB1R involvement, as the timescale of HRD-dependent short-term facilitation (10-20 Hz) is incompatible with CB1 signaling kinetics; the more likely mechanism is altered vesicle replenishment during high-frequency activity. (8) In the abstract, the long sentence beginning with "However, loss of the HRD..." should be divided for clarity. (9) The authors should ensure that all extended data, including mass spectrometry datasets, are deposited in an appropriate public repository. (10) Finally, conclusions regarding sample-size sufficiency and behavioral null results should be tempered, in agreement with concerns raised by multiple reviewers. Addressing these points will substantially strengthen the manuscript and ensure that the conclusions are well supported.
