# Supplementary figures and images for: The Neurexin1β Histidine-Rich Domain Is Involved in Excitatory Presynaptic Organization and Short-Term Plasticity
Source: eNeuro. 2026 Jan 30;13(2):ENEURO.0399-25.2026. doi: 10.1523/ENEURO.0399-25.2026 (PMC12894811; doi:10.1523/ENEURO.0399-25.2026)

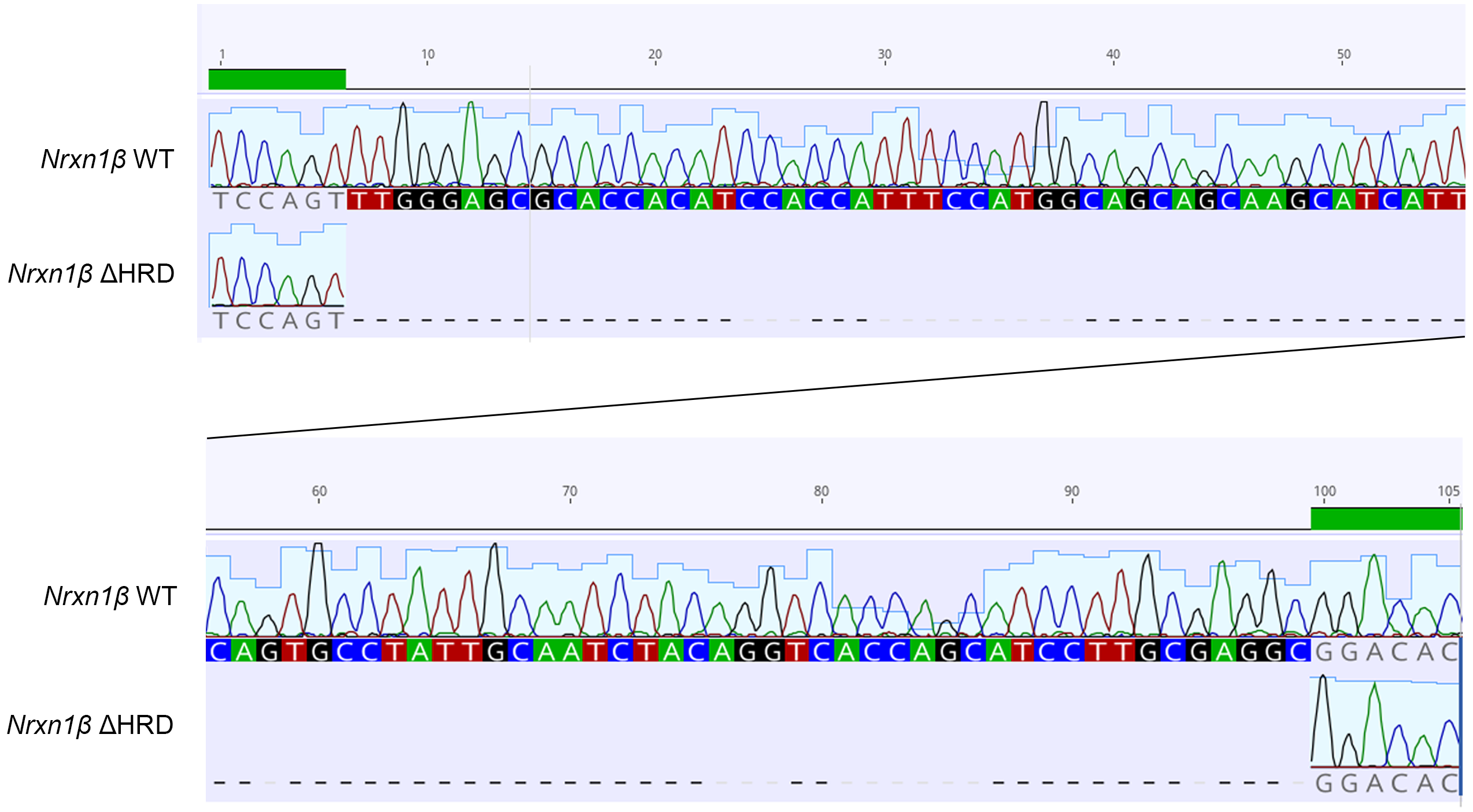

Supplement: Figure 1-1 — Confirmation of the mouse line in-frame Nrxn1β HRD deletion through sequencing Following genotyping, the WT and ΔHRD bands from a heterozygous Nrxn1β ΔHRD mouse (see Fig. 1c) were excised from the agarose gel and cloned into a plasmid for sequencing. Sequencing confirmed the deletion of the 93 bp region (highlighted bases) encoding the HRD in the mature protein. Download Figure 1-1, TIF file. [file eneuro-13-ENEURO.0399-25.2026-s001.tif]

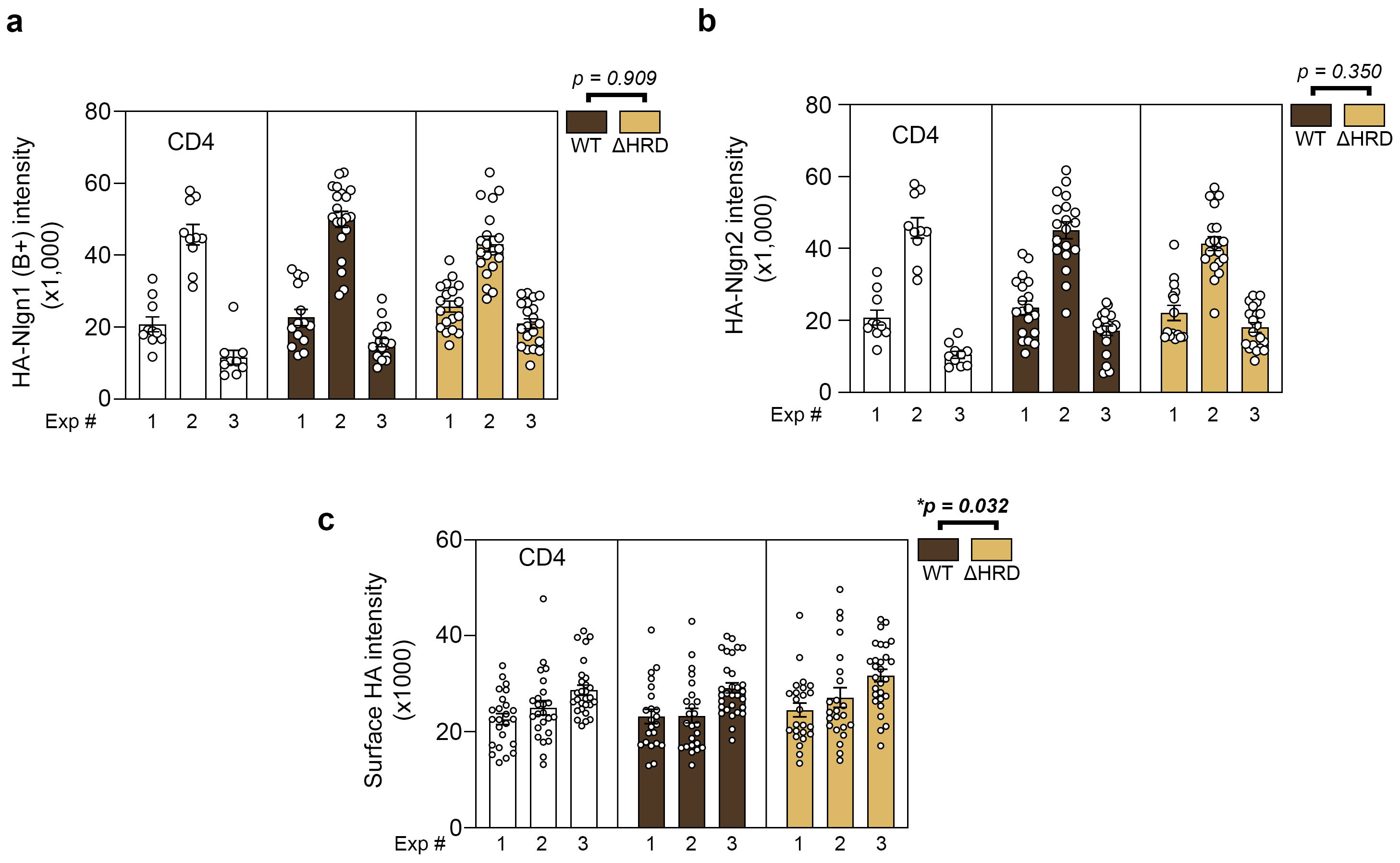

Supplement: Figure 3-1 — Quantification of surface expression levels of the extracellular HA-tagged constructs (a – b) Quantification of the average intensity of surface HA on HEK293 T cells expressing either HA-Nlgn1 (B+) or HA-Nlgn2 used in Fig. 3. Statistical significance was assessed using a linear mixed-effects model. Data are presented as mean ± SEM. n ≥ 9 cells for each experiment. (c) Quantification of surface expression intensity of HA-tagged CD4, Nrxn1β and Nrxn1βΔHRD. Nrxn1βΔHRD surface expression appears slightly higher than that of the WT protein with an estimated increase of 2570 units (95% CI: [227–4914]), which is small compared to the within-experiment variability. Therefore, we consider the expression level comparable. Statistical significance was assessed using a linear mixed-effects model. Data are presented as mean ± SEM. n ≥ 22 cells for each experiment. Download Figure 3-1, TIF file. [file eneuro-13-ENEURO.0399-25.2026-s002.tif]

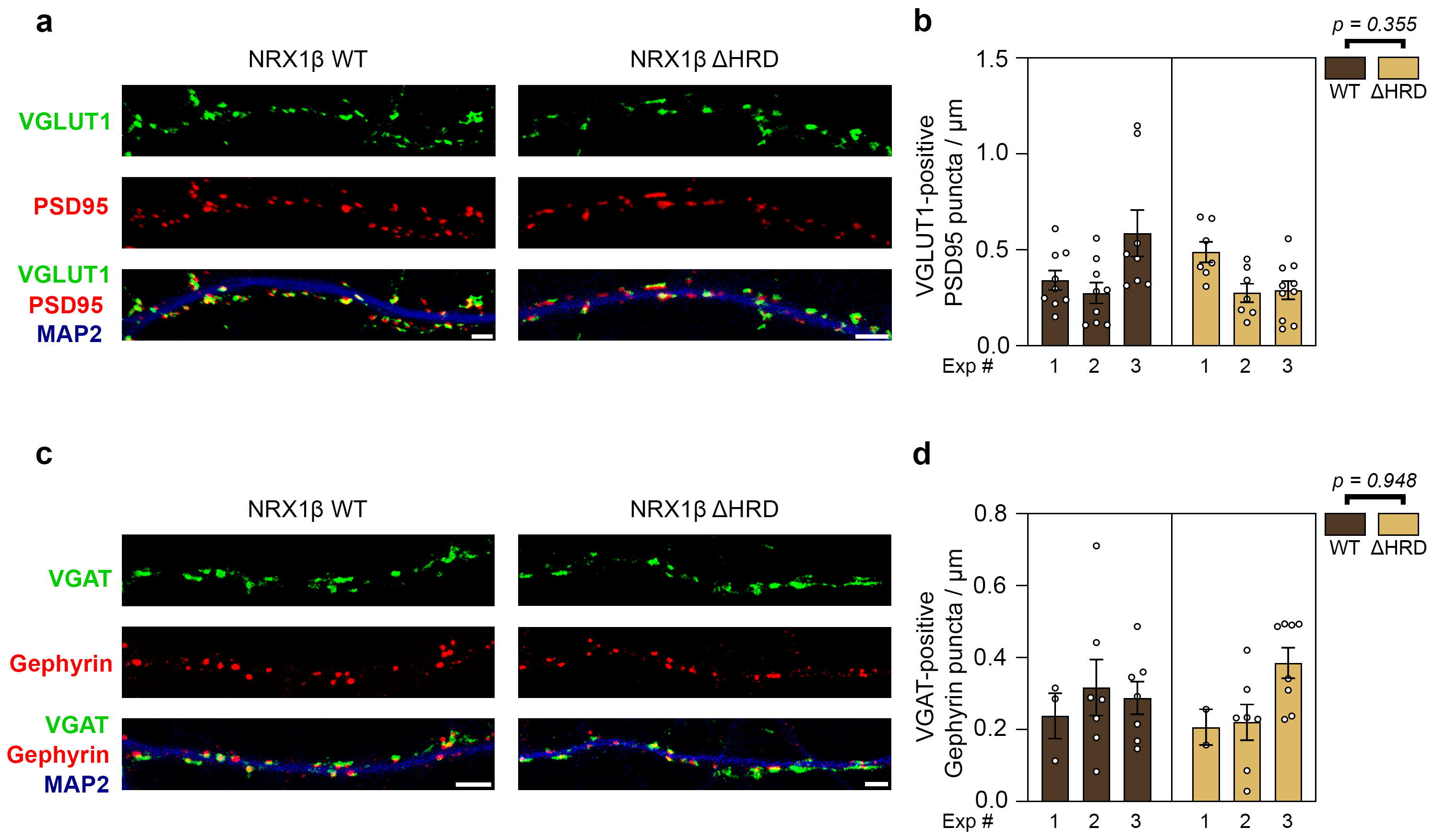

Supplement: Figure 3-2 — Comparison of excitatory and inhibitory synapse density in primary cortical neurons from Nrxn1β ΔHRD and WT mice (a) Representative images of Nrxn1β WT or ΔHRD-expressing neurons immunostained for VGLUT1 (green) and PSD95 (red) to mark excitatory pre- and post-synaptic compartments, respectively. MAP2 (blue) is used in all conditions as a marker of dendrites. Scale bar: 2 µm. (b) Quantification of VGLUT1-positive PSD95 puncta (representative of excitatory synapses) per micrometer on secondary dendrites. Statistical significance was assessed using a linear mixed-effects model. Data are presented as mean ± SEM. n = 3 independent experiments (at least 7 images per experiment were analyzed). (c) Representative images of Nrxn1β WT or ΔHRD-expressing neurons immunostained for VGAT (green) and gephyrin (red) to mark inhibitory pre- and post-synaptic compartments, respectively. MAP2 (blue) is used in all conditions as a marker of dendrites. Scale bar: 2 µm. (d) Quantification of VGAT-positive gephyrin puncta (representative of inhibitory synapses) per micrometer on secondary dendrites. Statistical significance was assessed using a linear mixed-effects model. Data are presented as mean ± SEM. n = 3 independent experiments (at least 7 images per experiment were analyzed). Download Figure 3-2, TIF file. [file eneuro-13-ENEURO.0399-25.2026-s003.tif]
